# Early Alzheimer´s disease blood biomarkers are associated with a higher risk for postoperative long‐term cognitive decline: Insights from the FINDERI study

**DOI:** 10.1002/alz.71631

**Published:** 2026-07-14

**Authors:** Niels Hansen, Monika Sadlonova, Hermann Esselmann, Christopher M. Celano, Carlotta Derad, Thomas Asendorf, Maike Hohberg, Mohammed Chebbok, Stephanie Heinemann, Barbara Morgado, Tobias Titsch, Katharina Packroß, Clara Maria Knopp, Matilda‐Marie Becker, Irina Günther, Iryna Krasiuk, Alina Isabel Rediske, Nicholas Paul Süttmann, Paul Thomas Itting, Oliver Wirths, Ingo Kutschka, Hassina Baraki, Christine A. F. von Arnim, Jens Wiltfang, FINDERI investigators

**Affiliations:** ^1^ Department of Psychiatry and Psychotherapy University Medical Center Göttingen Georg‐August‐University Göttingen Germany; ^2^ Department of Psychosomatic Medicine and Psychotherapy University Medical Center Göttingen Georg‐August‐University Göttingen Germany; ^3^ Department of Cardiovascular and Thoracic Surgery University Medical Center Göttingen Georg‐August‐University Göttingen Germany; ^4^ Department of Geriatrics University Medical Center Göttingen Georg‐August‐University Göttingen Germany; ^5^ German Center for Cardiovascular Research (DZHK) partner site Lower Saxony Germany; ^6^ Department of Psychiatry Massachusetts General Hospital Boston Massachusetts USA; ^7^ Department of Psychiatry Harvard Medical School Boston Massachusetts USA; ^8^ Department of Medical Statistics University Medical Center Göttingen Georg‐August‐University Göttingen Germany; ^9^ Department of Cardiology and Pneumology University Medical Center Göttingen Georg‐August‐University Göttingen Germany; ^10^ University Heart Center Basel University Hospital Basel Basel Switzerland; ^11^ German Center for Neurodegenerative Diseases (DZNE) Göttingen Germany; ^12^ Department of Medical Sciences Neurosciences and Signaling Group Institute of Biomedicine (iBiMED) University of Aveiro Aveiro Portugal

**Keywords:** Alzheimer´s disease, blood biomarker, postoperative cognitive dysfunction

## Abstract

**BACKGROUND:**

The study aim is to investigate whether blood biomarkers (BBMs) of Alzheimer's disease (AD) pathology are associated with postoperative cognitive dysfunction (POCD) after cardiac surgery.

**METHODS:**

Cognitive performance was assessed before and 12 months postoperatively using the Montreal Cognitive Assessment (MoCA) and categorized into stages—minimal (1), notable (2), and substantial (3) decline—in the FIND DElirium RIsk factors (FINDERI) study of patients undergoing cardiac surgery. BBMs were measured preoperatively (amyloid beta [Aβ]1‐42, Aβ1‐40, phosphorylated tau 181 [p‐tau181], p‐tau217, apolipoprotein E ε4 [apoE4] and apoE).

**RESULTS:**

A total of 394 patients completed follow‐up investigations. POCD Stage 1 was observed in 105 (26.6%), POCD Stage 2 in 52 patients (13.2%), and POCD Stage 3 in 30 patients (7.6%). The AT^217^term (ratio Aβ1‐40/1‐42 * p‐tau217) was significantly associated with POCD stages in multiple logistic regression.

**DISCUSSION:**

Early Alzheimer's BBMs are associated with POCD in patients, suggesting that our exploratory findings assessing BBMs may support risk stratification, inform decision‐making, and contribute to strategies aimed at preventing POCD.

## Highlights

- Postoperative cognitive dysfunction is a frequent condition after cardiac surgery
- Blood‐based Alzheimer's traits predict in‐sample postoperative cognitive dysfunction
- Blood biomarkers of Alzheimer's disease may improve preoperative risk stratification
- We relied on the AT^217^term hybrid ratio to detect early Alzheimer's pathology

## 1 BACKGROUND

Postoperative cognitive dysfunction (POCD) is a frequent complication following cardiac surgery affecting cognitive domains,^1^, ^2^ and it is often associated with postoperative delirium (POD).^3^, ^4^ Increasing evidence suggests that biomarkers indicative of Alzheimer's disease (AD) pathology in cerebrospinal fluid (CSF) or in the blood may represent a key determinant of the POCD risk in older surgical patients.^5^, ^6^, ^7^, ^8^, ^9^, ^10^ AD pathology develops years before the onset of clinical symptoms, and can be detected through CSF or blood‐based biomarker (BBM) signatures.^11^, ^12^ Consistent with this concept, reduced plasma amyloid beta (Aβ)42 peptide levels have been observed in patients who develop POCD,^10^ supporting a link between blood‐based AD pathology and the occurrence of POCD within a short postoperative interval. Recent advances in high‐throughput BBM platforms have enabled sensitive and reliable detection of cerebral β‐amyloidosis.^13^, ^14^, ^15^, ^16^, ^17^, ^18^ However, the evidence on the predictive value of AD biomarkers for POCD remains inconclusive. A recent CSF‐based meta‐analysis reported that reduced Aβ peptide levels were associated with POCD occurrence, whereas phosphorylated tau 181 (p‐tau181) and the Aβ42/t‐tau ratio did not show significant associations.^19^ P‐tau181 is a well‐known marker of Alzheimer's pathology, but in this study, we are specifically investigating biomarkers of early Alzheimer's pathology such as p‐tau217 and Aβ1‐42/1‐40. In 82 preselected patients with early AD, we demonstrated that the hybrid ratio AT^217^term is more diagnostically accurate (area under the curve [AUC] 0.96) than p‐tau217 alone (AUC 0.94) or Aβ42/40 (AUC: 0.91).^15^ The advantage of the AT^217^term over other hybrid ratios such as Aß42/40 is that its effect size is greater than that of p‐tau217, and that it possesses high diagnostic accuracy in predicting cerebral β‐amyloidosis.^15^ We therefore investigated whether BBMs of early AD pathology can predict the primary outcome POCD after primary exposure to cardiac surgery by relying on markers such as p‐tau217, Aβ1‐42/Aβ1‐40 ratio, and the novel hybrid ratio AT^217^term in patients undergoing cardiac surgery.

**RESEARCH IN CONTEXT**

1. **Systematic review**: We searched PubMed for the terms “blood biomarker,” “Alzheimer's disease,” and “postoperative cognitive dysfunction,” in order to discuss our findings in relation to the evidence from the most recent literature.
2. **Interpretation**: In a follow‐up examination of 394 patients 12 months after cardiac surgery, we have demonstrated that the AT^217^term (ratio Aβ1‐40/1‐42*p‐tau217) is an important in‐sample predictor of different stages of postoperative cognitive dysfunction (POCD) revealing varying degrees of cognitive impairment severity. Our results show that the blood biomarkers (BBMs) of Alzheimer's pathology may facilitate risk stratification, inform decision‐making, and contribute to strategies aimed at preventing POCD.
3. **Future directions**: Our findings need to be verified in larger cohorts that will enable us to test the reliability of our findings. The strength of our most recent observations lies in the fact that, according to validation studies, integrating BBM into routine preoperative screening could contribute to better‐tailored perioperative management strategies aiming to minimize POCD.

## 2 METHODS

We prospectively recruited *n* = 504 patients with various indications scheduled for cardiac surgery during the years 2021–2022, the primary exposure in this analysis. POCD was defined as the primary outcome to investigate the effect of BBMs on 12‐month POCD. POD was considered a secondary study endpoint of this analysis. Study participants were enrolled in the Department of Cardiovascular and Thoracic Surgery at the University Medical Center Göttingen, based on the following inclusion criteria: age ≥50 years, hospitalization for elective cardiac surgery, ability to speak German, and absence of diagnosed dementia. All patients provided written informed consent. The study was conducted in accordance with the Declaration of Helsinki and approved by the ethics committee in Göttingen (AZ20/11/20, amendment 21/07/21). The study was registered in the German Clinical Trials Register (DRKS00025095). The biological samples in our biobank were collected and stored in accordance with the ethics approval for biobanking (AZ9/2/2016).

### 2.1 Biobanking

Blood samples for biomarker analyses were collected 1–2 days prior to surgery. All samples were processed according to a standardized protocol and stored in the Biobank at the Department of Psychiatry and Psychotherapy, Göttingen. Ethylenediaminetetraacetic acid (EDTA) plasma was centrifuged at 2000 x *g* for 10 min. The resulting plasma was aliquoted into 500 µL portions and stored at −80°C until further use.

### 2.2 Cognitive assessment

The influence of BBMs on our primary outcome, namely 12‐month POCD after cardiac surgery, is investigated in this study. Cognitive performance was measured at baseline and at 12‐month follow‐up after surgery via the Montreal Cognitive Assessment (MoCA).^20^ At the 1‐year follow‐up, participants completed either the MoCA or the telephone‐adapted t‐MoCA; t‐MoCA scores (0–22) were converted to the 30‐point MoCA scale using equipercentile equation.^21^ POCD was defined using a new graduated decline approach considering previous gradual POCD definitions.^22^ As demonstrated previously,^23^ different cutoff values of MoCA can be applied in research settings to better capture a gradual decline in cognitive performance. Furthermore, the cutoff value of 23 is particularly well suited to detect mild cognitive impairment (MCI), as this value revealed superior diagnostic accuracy among the various examined parameters.^24^ There is also evidence that this cutoff value is particularly suitable for determining whether MCI is more advanced, as patients in one study^25^ were already in the dementia stage at the cutoff value of 21. Thus, in addition to the generally recommended cutoff value of 26, useful cutoff values are 21 and 23 for gradually differentiating POCD and thus achieving a graded classification of POCD. It is structured as a gradual POCD approach, as the minimal change relative to the general cut‐point of 26, 23, and 21, respectively. According to the literature, the concept of gradual decline encompasses additional cutoff points (namely, 23^24^ and 21^25^). This enables a graded assessment of the cutoff points for MCI, such as POCD. A decline in MoCA score from ≥26 at baseline to <26 at 12 months, or any further decrease if baseline was already <26, was classified as a POCD Stage 1 (minimal decline). The same logic applied to MoCA thresholds of 23 (Stage 2 POCD) and 21 (Stage 3 POCD), enabling a graduated procedure for POCD assessment. These various cutoff points refer to an individual's cognitive status 12 months after heart surgery (for more details see supplement text). In addition, patients were asked whether they had experienced subjective cognitive decline (SCD) prior to surgery and 1 year after surgery. POD was assessed on postoperative Days 1 to 5 via the Confusion Assessment Method (CAM)^26^ test applied twice daily.

### 2.3 Measuring blood biomarkers

Plasma concentrations of p‐tau181, Aβ1‐42, and Aβ1‐40 were quantified from EDTA plasma samples using Fujirebio's fully automated Lumipulse G600 II platform. Measurements were performed with Lumipulse G pTau 181 plasma (product numbers 81288, 81289), Lumipulse G β‐amyloid 1‐42 plasma (product numbers 81301, 81303), and Lumipulse G β‐amyloid 1‐40 plasma (product numbers 231524, 231531). Plasma p‐tau217 concentrations were measured using the S‐Plex immunoassay (Meso Scale Diagnostics [MSD]). Based on these measurements, we calculated the following hybrid ratios: (1) Aβ1‐42/ 1‐40 ratio, (2) AT^217^term (ratio Aβ1‐40/1‐42 * p‐tau217) and (3) AT181term (ratio Aβ1‐40/1‐42 * p‐tau181). Apoliprotein E4 (ApoE4) and Apoliprotein E (ApoE) as protein expressions were measured by Lumipulse immunoassays (Lumipulse G ApoE, product number 81452, Lumipulse G APOEe4 allele (ApoE4) product number, 81453). In addition, the ApoE4 genotype was determined from whole‐blood samples using real‐time polymerase chain reaction (PCR) according to a published protocol.^27^

### 2.4 Statistical approach

All statistical analyses were performed in R version 4.4.3. Continuous variables, including BBMs, are summarized as mean ± standard deviation (SD), and categorical variables as counts and percentages (*n*, %). Differences between patients without and with POCD across the increasing levels of cognitive impairment severity (POCD Stages 1, 2, and 3) were assessed using the Welch two‐sample *t*‐test for continuous variables and Pearson's chi‐square test for categorical variables, with Fisher's exact test applied when expected cell counts were low. In addition, receiver‐operating characteristic (ROC) curve analyses were performed, with area under the curve (or AUC) reported with logit‐transformed, permutation based 95% confidence intervals (CIs). Cutoff values for the biomarkers in patients with and without POCD states were determined by simultaneous maximization of sensitivity and specificity. BBMs were z‐transformed with respect to the total population for regression analysis. Univariate regression analyses were conducted to assess BBMs as explaining variables of the POCD stages. Furthermore, multiple logistic regression models were examined to evaluate BBMs and clinical factors, including sex, age, body mass index (BMI), renal failure, coronary artery bypass grafting (CABG), valve surgery, other surgery, surgery duration, and POD, as predictors for POCD. We examined a total of six distinct models to identify the best‐fitting combination of clinical parameters and BBMs. Model 1 contained the clinical parameters listed above and the BBMs Aβ1‐42, Aβ1‐40, p‐tau217, and p‐tau181. In Models 2–4, the clinical parameters were combined with specific derived BBM ratios (Aβ1‐42/1‐40 ratio, AT^181^term, AT^217^term). Model 5 included only the clinical variables, and Model 6 only the BBMs Aβ1‐42, Aβ1‐40, p‐tau217, and p‐tau181. Model selection was based on the Akaike information criterion (AIC) and Bayesian information criterion (BIC), with the model exhibiting the lowest values considered the optimal balance between model complexity and goodness of fit. To assess multicollinearity, the variance inflation factor (VIF) was calculated, with values >5 indicating moderate collinearity.^28^ When integrating ApoE4 genotype and proteotype into the models, collinearity was assessed using the generalized VIF (GVIF). GVIF values were adjusted according to the formula GVIF^ [1/(2 × df)] to allow comparison across explaining variables with differing degrees of freedom. In addition, we adjusted the MoCA score at 12 months for the baseline value in univariate and multiple regression analyses to assess risk factors of POCD via another approach. We also took a marginal effects approach to investigate the potential association between POD and POCD, thus enabling us to analyze our data without the strict assumptions of a mediation model while still testing whether patients with POD carry a higher risk of developing POCD. We used a logistic regression model to assess the marginal effects of POD and the AT^217^ term on the risk at each stage of POCD. Statistical significance was set at two‐sided *p*  <  0.05.

## 3 RESULTS

### 3.1 Clinical parameters in patients with POCD

A total of 504 patients were recruited in the FIND DElirium RIsk factors (FINDERI) study, of whom 394 completed cognitive assessment 1 year after surgery and were evaluated for the presence of POCD. Of these, 105 patients (26.6%) met criteria for POCD Stage 1 with a mean MoCA score of 21.77 ± 3.21, 1 year after surgery; 52 (13.2%) for moderate POCD Stage 2 with a mean MoCA score of 19.31 ± 2.84, 1 year after surgery; and 30 (7.6%) for severe POCD Stage 3 with a mean MoCA score of 17.67 ± 2.57, 1 year after surgery (Table 1). We found no statistically significant differences in patients with SCD at baseline and after 1 year across POCD stages (Table 1). Only patients with POCD Stage 3 were, on average, older than those without POCD Stage 3 (Table 1). Other clinical variables, including sex, BMI, renal function, diabetes, type of surgery, POD, and duration of surgery, did not reveal consistent differences across POCD stages (Table 1).

**TABLE 1:** POCD–Clinical parameter and blood biomarkers.

	Complete cohort	With POCD‐Status	POCD Stage 1			POCD Stage 2			POCD Stage 3		
Characteristic	*N* = 504^a^	*N* = 394^a^	No POCD *N* = 289^a^	POCD *N* = 105^a^	*p*‐value^b^	No POCD N = 342^a^	POCD *N* = 52^a^	*p*‐value^b^	No POCD *N* = 364^a^	POCD *N* = 30^a^	*p*‐value^b^
1y‐FU MoCA (0–30)	24.83 ± 3.46	24.83 ± 3.46	25.94 ± 2.83	21.77 ± 3.21		25.67 ± 2.69	19.31 ± 2.84		25.43 ± 2.78	17.57 ± 2.57	
*N*	394	394									
Baseline MoCA (0–30)	23.83 ± 3.62	24.16 ± 3.34	24.05 ± 3.47	24.49 ± 2.94	0.215	24.43 ± 3.35	22.42 ± 2.72	<0.001	24.43 ± 3.26	20.93 ± 2.52	<0.001
*N*	499	394									
Baseline MoCA category					0.109			<0.001			<0.001
<21	83 (16.6%)	51 (12.9%)	38 (13.1%)	13 (12.4%)		38 (11.1%)	13 (25.0%)		38 (10.4%)	13 (43.3%)	
21–22	81 (16.2%)	63 (16.0%)	52 (18.0%)	11 (10.5%)		52 (15.2%)	11 (21.2%)		52 (14.3%)	11 (36.7%)	
23–25	143 (28.7%)	124 (31.5%)	94 (32.5%)	30 (28.6%)		103 (30.1%)	21 (40.4%)		120 (33.0%)	4 (13.3%)	
≥26	192 (38.5%)	156 (39.6%)	105 (36.3%)	51 (48.6%)		149 (43.6%)	7 (13.5%)		154 (42.3%)	2 (6.67%)	
*N*	499	394									
Baseline SCD	224 (45.3%)	176 (45.2%)	124 (43.2%)	52 (51.0%)	0.215	149 (44.0%)	27 (54.0%)	0.238	159 (44.2%)	17 (58.6%)	0.190
*N*	495	389	287	102		339	50		360	29	
1y‐FU SCD	141 (35.6%)	140 (35.5%)	98 (33.9%)	42 (40.0%)	0.318	118 (34.5%)	22 (42.3%)	0.347	126 (34.6%)	14 (46.7%)	0.260
*N*	396	394									
Age, years	68.3 ± 8.2	68.1 ± 8.2	68.1 ± 8.0	68.3 ± 8.8	0.785	67.8 ± 8.1	70.0 ± 8.3	0.078	67.8 ± 8.1	72.0 ± 8.0	0.009
Sex					0.279			0.061			0.221
Male	396 (78.6%)	314 (79.7%)	226 (78.2%)	88 (83.8%)		267 (78.1%)	47 (90.4%)		287 (78.8%)	27 (90.0%)	
Female	108 (21.4%)	80 (20.3%)	63 (21.8%)	17 (16.2%)		75 (21.9%)	5 (9.62%)		77 (21.2%)	3 (10.0%)	
BMI	28.27 ± 4.72	28.40 ± 4.69	28.35 ± 4.61	28.53 ± 4.92	0.748	28.31 ± 4.60	28.96 ± 5.28	0.403	28.42 ± 4.62	28.18 ± 5.55	0.825
*N*	496	390	285	105		338	52		360	30	
Renal failure	64 (12.7%)	48 (12.2%)	37 (12.8%)	11 (10.5%)	0.653	40 (11.7%)	8 (15.4%)	0.596	44 (12.1%)	4 (13.3%)	0.774
*N*	503	394									
Diabetes	151 (30.0%)	111 (28.2%)	86 (29.8%)	25 (23.8%)	0.301	99 (28.9%)	12 (23.1%)	0.477	104 (28.6%)	7 (23.3%)	0.688
CABG	329 (65.3%)	263 (66.8%)	200 (69.2%)	63 (60.0%)	0.111	234 (68.4%)	29 (55.8%)	0.100	246 (67.6%)	17 (56.7%)	0.309
Valve surgery	230 (45.6%)	171 (43.4%)	123 (42.6%)	48 (45.7%)	0.657	146 (42.7%)	25 (48.1%)	0.562	158 (43.4%)	13 (43.3%)	>0.999
Other surgery	107 (21.2%)	80 (20.3%)	54 (18.7%)	26 (24.8%)	0.236	65 (19.0%)	15 (28.8%)	0.145	72 (19.8%)	8 (26.7%)	0.506
Surgery duration, h	4.37 ± 1.40	4.33 ± 1.36	4.37 ± 1.36	4.22 ± 1.38	0.341	4.39 ± 1.36	3.96 ± 1.31	0.032	4.37 ± 1.35	3.83 ± 1.44	0.058
POD	106 (21.6%)	73 (18.8%)	54 (18.9%)	19 (18.4%)	>0.999	62 (18.4%)	11 (21.6%)	0.728	63 (17.5%)	10 (34.5%)	0.046
*N*	491	388	285	103		337	51		359	29	
Aβ1‐40, pg/mL	401.02 ± 123.99	397.12 ± 124.01	395.07 ± 108.63	402.74 ± 159.29	0.654	392.26 ± 112.60	428.67 ± 179.84	0.166	393.61 ± 117.42	439.89 ± 184.05	0.193
*N*	483	382	285	102		331	51		353	29	
Aβ1‐42, pg/mL	31.12 ± 8.74	30.94 ± 8.70	30.76 ± 7.69	31.43 ± 11.02	0.578	30.63 ± 7.85	32.93 ± 12.82	0.220	30.78 ± 8.45	32.86 ± 11.31	0.341
*N*	483	382	280	102		331	51		353	29	
Ratio Aβ1‐42/Aβ1‐40	0.0786 ± 0.0100	0.0789 ± 0.0094	0.0788 ± 0.0095	0.0792 ± 0.0091	0.671	0.0791 ± 0.0096	0.0777 ± 0.0084	0.292	0.0791 ± 0.0094	0.0761 ± 0.0088	0.093
*N*	483	382	280	102		331	51		353	29	
p‐tau181, pg/mL	1.85 ± 2.87	1.81 ± 3.17	1.58 ± 0.92	2.44 ± 5.90	0.145	1.57 ± 0.91	3.36 ± 8.23	0.124	1.80 ± 3.29	1.99 ± 0.90	0.422
*N*	496	388	284	104		336	52		358	30	
AT^181^term	24.51 ± 40.86	23.97 ± 44.47	20.81 ± 12.89	32.62 ± 83.05	0.156	20.65 ± 12.88	45.49 ± 115.87	0.132	23.68 ± 46.10	27.40 ± 13.35	0.289
*N*	483	382	280	102		331	51		353	29	
p‐tau217, pg/mL	4.59 ± 4.68	4.35 ± 3.89	3.93 ± 2.83	5.51 ± 5.74	0.009	3.92 ± 3.02	7.18 ± 6.74	0.001	4.16 ± 3.63	6.68 ± 5.79	0.029
*N*	483	382	280	102		331	51		353	29	
AT^217^term	61.64 ± 71.01	57.61 ± 54.61	51.86 ± 39.29	73.39 ± 81.50	0.012	51.78 ± 43.72	95.42 ± 91.77	0.002	54.89 ± 51.07	90.74 ± 80.95	0.026
*N*	483	382	280	102		331	51		353	29	
p‐tau181/p‐tau217 ratio	0.45 ± 0.20	0.46 ± 0.19	0.45 ± 0.17	0.46 ± 0.24	0.715	0.46 ± 0.18	0.45 ± 0.29	0.787	0.46 ± 0.20	0.39 ± 0.18	0.061
*N*	483	382	280	102		331	51		353	29	
ApoE4 genotype					0.415			0.175			0.070
ApoE non‐e4	345 (71.9%)	271 (71.5%)	200 (71.9%)	71 (70.3%)		238 (72.6%)	33 (64.7%)		255 (72.9%)	16 (55.2%)	
Heterozygous e4	123 (25.6%)	98 (25.9%)	69 (24.8%)	29 (28.7%)		80 (24.4%)	18 (35.3%)		85 (24.3%)	13 (44.8%)	
Homozygous e4	12 (2.50%)	10 (2.64%)	9 (3.24%)	1 (0.99%)		10 (3.05%)	0 (0%)		10 (2.86%)	0 (0%)	
*N*	479	379	278	101		328	51		350	29	
Ratio ApoE4/ApoE	0.17 ± 0.38	0.18 ± 0.39	0.19 ± 0.41	0.16 ± 0.31	0.562	0.18 ± 0.41	0.15 ± 0.22	0.407	0.18 ± 0.40	0.20 ± 0.25	0.627
*N*	482	381	279	102		330	51		352	29	
ApoE, µg/mL	19.66 ± 12.84	19.52 ± 12.93	18.92 ± 12.40	21.16 ± 14.22	0.161	19.29 ± 12.77	20.99 ± 13.96	0.417	19.43 ± 12.80	20.63 ± 14.61	0.671
*N*	482	381	279	102		330	51		352	29	
ApoE4, µg/mL	2.53 ± 7.17	2.65 ± 7.60	2.76 ± 8.47	2.33 ± 4.44	0.522	2.71 ± 8.05	2.21 ± 3.53	0.446	2.61 ± 7.82	3.07 ± 4.15	0.600
*N*	482	381	279	102		330	51		352	29	

Abbreviations: 1y‐FU, 1‐year follow‐up investigation; 1y‐FU SCD, 1‐year follow‐up with subjective decline; Aβ1‐42, amyloid beta 1‐42; Aβ1‐40, amyloid beta 1‐40; ApoE4, apoliprotein E allele 4; ApoE, apolipoprotein E; AT^181^term, Aβ1‐40/Aβ1‐42*p‐tau181; AT^217^term, Aβ1‐40/Aβ1‐42*p‐tau217; BMI, body mass index; CABG, coronary bypass graft; h, hours; MoCA, Montreal Cognitive Assessment; *N*, number of available participants; POD, postoperative delirium; POCD, postoperative cognitive dysfunction; p‐tau181, phosphorylated tau protein 181; p‐tau217, phosphorylated tau protein 217; SCD, subjective cognitive decline.

^a^ *n* (%); mean ± SD.

^b^ Welch two‐sample *t*‐test for continuous variables and Pearson's chi‐square test resp. Fisher's exact test in cases of low expected cell counts for categorical variables.

### 3.2 BBMs of early Alzheimer's disease in patients with POCD

**FIGURE 1:**
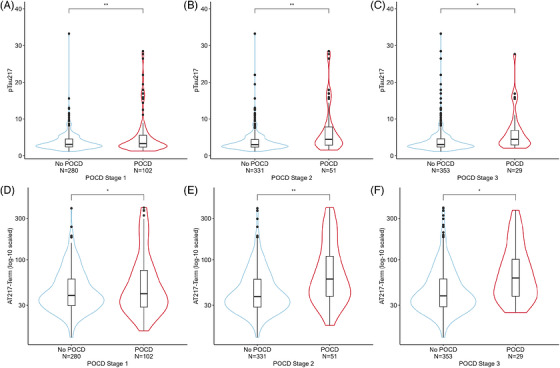
Blood biomarkers p‐tau217 and AT^217^term differ between patients with POCD compared to those without POCD. The p‐tau217 is significantly higher in the blood of patients with POCD Stage 1 (A), POCD Stage 2 (B), and POCD Stage 3 (C) POCD in A, B, and C compared to patients without POCD Stages 1, 2, and 3. In addition, AT^217^term is also significantly higher in the blood of patients with POCD Stage 1 (D), POCD Stage 2 (E), and POCD Stage 3 (F) than in patients without mild, moderate, and severe POCD. Abbreviations: AT^217^term, ratio Aβ1‐42/1‐40 * p‐tau217; p‐tau217, phosphorylated tau protein 217.

Plasma levels of p‐tau217 were significantly higher in patients exhibiting any POCD stage than those without POCD (Table 1, Figure 1). Similarly, the AT^217^term biomarker was consistently higher across all POCD stages relative to no POCD (Table 1, Figure 1). In contrast, we observed no statistically significant differences between patients with or without POCD in preoperative levels of the other investigated BBMs (Table 1). In addition, there were no differences between POCD stages after stratification according to the ApoE genotype or the ApoE proteotype (Table S1).

### 3.3 ROC analysis and optimal cutoff values for BBM

ROC curve analysis was performed to evaluate the in‐sample discriminative ability of each biomarker at the POCD stages (Table 2, Table S2). None of the BBMs investigated demonstrated statistically significant discriminative ability for POCD Stage 1 (Table 2, Table S2). However, AT^181^term, p‐tau217, and AT^217^term demonstrated significant discrimination between patients with and without POCD Stage 2, with moderate AUCs (AT^181^term: AUC = 0.675, 95% CI 0.5897–0.7517, *p* < 0.001; p‐tau217: AUC = 0.679, 95% CI 0.588–0.755, *p* < 0.001; AT^217^term: AUC = 0.679, 95% CI 0.593–0.756, *p* < 0.001; Table 2 with optimal cutoff values). For POCD Stage 3, several BBMs showed moderate, but significant discriminative ability (Table 2), that is, the Aβ1‐42/1‐40 ratio (AUC = 0.615, 95% CI 0.509–0.713, *p* = 0.036), AT^217^term (AUC = 0.692, 95% CI 0.587–0.778, *p* < 0.001), AT^181^term (AUC = 0.675, 95% CI 0.564–0.770, *p* = 0.002), and p‐tau217 (AUC = 0.685, 95% CI 0.578–0.777, *p* = 0.001). Taken together, AT^217^term, AT^181^term, and p‐tau217 emerged as BBMs with moderate discriminative ability for POCD Stages 2 and 3 but not Stage 1.

**TABLE 2:** Area under the curves and optimal cutoffs for 12‐month POCD.

Variable	AUC (95% CI)	*p*‐value	Optimal cutoff	Sensitivity (95% CI)	Specificity (95% CI)	PPV (95% CI)	NPV (95% CI)
**POCD: Stage 1**							
Aβ1‐42/1‐40 ratio	0.5079 (0.4409, 0.5734)	0.817	<0.0806	0.5098 (0.4089, 0.6101)	0.5321 (0.4718, 0.5918)	0.2842 (0.2201, 0.3554)	0.7487 (0.6825, 0.8074)
AT^181^term	0.5501 (0.4795, 0.6179)	0.166	<18.1508	0.5294 (0.428, 0.629)	0.5429 (0.4825, 0.6023)	0.2967 (0.2314, 0.3688)	0.76 (0.6947, 0.8174)
p‐tau217	0.5348 (0.4649, 0.6022)	0.328	<3.2615	0.5294 (0.428, 0.629)	0.5393 (0.479, 0.5988)	0.2951 (0.2301, 0.3668)	0.7588 (0.6932, 0.8165)
AT^217^term	0.5302 (0.4591, 0.5978)	0.404	<39.9812	0.5294 (0.428, 0.629)	0.5286 (0.4683, 0.5883)	0.2903 (0.2262, 0.3612)	0.7551 (0.6887, 0.8136)
ApoE4/ApoE ratio	0.5064 (0.455, 0.5627)	0.815	<0.21	0.3137 (0.2255, 0.4131)	0.7168 (0.6601, 0.7689)	0.2883 (0.2063, 0.382)	0.7407 (0.6841, 0.792)
ApoE4	0.5303 (0.4729, 0.5881)	0.310	<0.1	0.4706 (0.371, 0.572)	0.6022 (0.5421, 0.66)	0.3019 (0.2317, 0.3796)	0.7568 (0.6948, 0.8117)
**POCD: Stage 2**							
Aβ1‐42/1‐40 ratio	0.5565 (0.4754, 0.6358)	0.178	>0.0789	0.549 (0.4034, 0.6887)	0.574 (0.5188, 0.6279)	0.1657 (0.113, 0.2305)	0.892 (0.8424, 0.9303)
AT^181^term	0.675 (0.5897, 0.7517)	<0.001	<20.6209	0.6471 (0.5007, 0.7757)	0.6465 (0.5924, 0.698)	0.22 (0.1565, 0.2949)	0.9224 (0.8802, 0.9534)
p‐tau217	0.6789 (0.5878, 0.7552)	<0.001	<3.4667	0.6275 (0.4808, 0.7587)	0.6133 (0.5585, 0.666)	0.2 (0.141, 0.2704)	0.9144 (0.8696, 0.9477)
AT^217^term	0.6792 (0.5926, 0.7557)	<0.001	<44.6606	0.6078 (0.4611, 0.7416)	0.6254 (0.5708, 0.6777)	0.2 (0.1401, 0.2717)	0.9119 (0.8672, 0.9454)
ApoE4/ApoE ratio	0.5194 (0.4562, 0.5955)	0.551	<0.21	0.3529 (0.2243, 0.4993)	0.7182 (0.6663, 0.7661)	0.1622 (0.099, 0.2441)	0.8778 (0.8327, 0.9144)
ApoE4	0.5531 (0.4792, 0.6319)	0.156	<0.1	0.4706 (0.371, 0.572)	0.6022 (0.5421, 0.66)	0.3019 (0.2317, 0.3796)	0.7568 (0.6948, 0.8117)
**POCD: Stage 3**							
Aβ1‐42/1‐40 ratio	0.6146 (0.5085, 0.7127)	0.036	>0.0778	0.5862 (0.3894, 0.7648)	0.6119 (0.5589, 0.663)	0.1104 (0.0656, 0.1709)	0.9474 (0.9099, 0.9725)
AT^181^term	0.6748 (0.5641, 0.7696)	0.002	<20.6209	0.6552 (0.4567, 0.8206)	0.6289 (0.5762, 0.6794)	0.1267 (0.078, 0.1907)	0.9569 (0.9222, 0.9791)
p‐tau217	0.6848 (0.5777, 0.7768)	0.001	<3.6407	0.6207 (0.4226, 0.7931)	0.6232 (0.5704, 0.674)	0.1192 (0.0722, 0.1818)	0.9524 (0.9164, 0.976)
AT^217^term	0.692 (0.5869, 0.7779)	0.001	<43.1277	0.6207 (0.4226, 0.7931)	0.5921 (0.5388, 0.6438)	0.1111 (0.0672, 0.1699)	0.95 (0.9123, 0.9748)
ApoE4/ApoE ratio	0.5721 (0.4871, 0.6799)	0.097	<0.21	0.4483 (0.2645, 0.6431)	0.7216 (0.6716, 0.7678)	0.1171 (0.0639, 0.1919)	0.9407 (0.9055, 0.9658)
ApoE4	0.6108 (0.5146, 0.7133)	0.025	<0.1	0.4706 (0.371, 0.572)	0.6022 (0.5421, 0.66)	0.3019 (0.2317, 0.3796)	0.7568 (0.6948, 0.8117)

*Note*: < values below cutoff are classified as healthy; > values above cutoff are classified as healthy; CI = confidence interval (exact binomial confidence limits).

Abbreviations: Aβ1‐42, amyloid beta 1‐42; Aβ1‐40, amyloid beta 1‐40; ApoE4, Apoliprotein E4; ApoE, Apoliprotein E; AT^181^term, Aβ1‐40/1‐42*p‐tau181; AT^217^term, Aβ1‐40/1‐42*p‐tau217; AUC, area under the curve with logit‐transformed; permutation based 95% confidence interval; NPV, negative predictive value; POCD, postoperative cognitive dysfunction; PPV, positive predictive value; p‐tau181, phosphorylated tau protein 181; p‐tau217, phosphorylated tau protein 217.

### 3.4 Univariate logistic regression for postoperative cognitive dysfunction

Univariate logistic regression analyses were conducted to evaluate the association between preoperative clinical variables and BBMs with POCD (Table 3 and Table S3). Among the BBMs assessed, p‐tau181 (*n* = 388, OR = 1.85, 95% CI 1.11–3.48, *p* = 0.039), p‐tau217 (*n* = 382, OR = 1.55, 95% CI 1.19–2.07, *p* = 0.002), AT^217^term (*n* = 382, OR = 1.58, 95% CI 1.20–2.13, *p* = 0.002), and AT^181^term (*n* = 382, OR = 1.85, 95% CI 1.10–3.46, *p* = 0.042) were significantly associated with an increased risk for POCD Stage 1 (Table 3). In addition, the biomarkers p‐tau181 (OR = 3.05, 95% CI 1.58–6.64, *p* = 0.003), the AT^181^term (OR = 3.18, 95% CI 1.62–6.74, *p* = 0.001), p‐tau217 (OR = 1.98, 95% CI 1.48–2.74, *p* < 0.001), and AT^217^term (OR = 3.18, 95% CI 1.62–6.74, *p* = 0.001) are significantly associated with POCD Stage 2 (Table 3). The clinical variables surgery duration (odds ratio [OR] = 0.77, 95% CI 0.60–0.97, *p* = 0.035) and female sex (OR = 0.38, 95% CI 0.13–0.90, *p* = 0.047) showed moderate associations with POCD Stage 2 (Table 3). Regarding POCD Stage 3, the BBM AT^217^term (OR = 1.66, 95% CI 1.18–2.30, *p* = 0.002), the ApoE4 (Apoliprotein E allele 4) heterozygous genotype (OR = 2.44, 95% CI 1.11–5.27, *p* = 0.024), and p‐tau217 (OR = 1.57, 95% CI 1.15–2.12, *p* = 0.003) were significant (Table 3). Furthermore, POD (OR = 2.47, 95% CI 1.06–5.47, *p* = 0.029) and older age (OR = 1.07, 95% CI 1.02–1.12, *p* = 0.008) were significantly associated with a higher risk for POCD Stage 3, whereas longer surgery duration (OR = 0.72, 95% CI 0.52–0.97, *p* = 0.036) resulted in a lower risk for POCD Stage 3 (Table 3). In addition, we adjusted the MoCA score at 12 months for the baseline value and found that the BBM values of p‐tau217 (β –1.0, 95% CI −1.4 to −0.62, *p* < 0.001) and AT^217^term (β −1.1, 95% CI −1.5 to −0.67, *p* < 0.001) were associated with a higher risk of POCD at 12 months (Table S4, see for other BBMs). Furthermore, taking this approach, we found that POD (β −1.7, 95% CI −2.6 to −0.85, *p* < 0.001) was associated with a higher risk of POCD at 12 months (Table S4, see for other clinical variables).

**TABLE 3:** Univariate logistic regressions in different stages of POCD.

	POCD Stage 1				POCD Stage 2				POCD Stage 3			
Characteristic	*N*	OR	95% CI	*p*‐value	*N*	OR	95% CI	*p*‐value	*N*	OR	95% CI	*p*‐value
Age	394	1.00	0.98, 1.03	0.775	394	1.03	1.00, 1.07	0.071	394	1.07	1.02, 1.12	0.008
Female sex	394	0.69	0.37, 1.23	0.223	394	0.38	0.13, 0.90	0.047	394	0.41	0.10, 1.21	0.156
Surgery duration, h	394	0.92	0.77, 1.09	0.336	394	0.77	0.60, 0.97	0.035	394	0.72	0.52, 0.97	0.036
POD	388	0.97	0.53, 1.70	0.911	388	1.22	0.57, 2.44	0.590	388	2.47	1.06, 5.47	0.029
Aβ1‐40 (SD)	382	1.06	0.84, 1.32	0.593	382	1.27	0.98, 1.63	0.057	382	1.32	0.96, 1.75	0.061
Aβ1‐42 (SD)	382	1.08	0.86, 1.34	0.511	382	1.25	0.96, 1.62	0.085	382	1.22	0.86, 1.66	0.219
p‐tau181 (SD)	388	1.85	1.11, 3.48	0.039	388	3.05	1.58, 6.64	0.003	388	1.04	0.63, 1.28	0.753
p‐tau217 (SD)	382	1.55	1.19, 2.07	0.002	382	1.98	1.48, 2.74	<0.001	382	1.57	1.15, 2.12	0.003
p‐tau181/p‐tau217 ratio (SD)	382	1.05	0.83, 1.31	0.671	382	0.94	0.67, 1.26	0.698	382	0.62	0.36, 1.00	0.069
Aβ1‐42/1‐40 ratio (SD)	382	1.05	0.83, 1.35	0.677	382	0.86	0.64, 1.17	0.335	382	0.73	0.50, 1.08	0.104
AT^181^term (SD)	382	1.85	1.10, 3.46	0.042	382	3.18	1.62, 6.74	0.001	382	1.05	0.66, 1.30	0.676
AT^217^term (SD)	382	1.58	1.20, 2.13	0.002	382	2.01	1.49, 2.81	<0.001	382	1.66	1.18, 2.30	0.002
ApoE‐e4 genotype^a^	379				379				379			
Heterozygous e4		1.18	0.70, 1.96	0.518		1.62	0.85, 3.01	0.131		2.44	1.11, 5.27	0.024
Homozygous e4		0.31	0.02, 1.71	0.275		0.00		0.985		0.00		0.991

Abbreviations: Aβ1‐42, amyloid beta 1‐42; Aβ1‐40, amyloid beta 40; ApoE, apolipoprotein E; AT^181^term, Aβ1‐40/Aβ1‐42*p‐tau181; AT^217^term, Aβ1‐40/Aβ1‐42*p‐tau217; CI, confidence interval; N, number; OR, odds ratio; p‐tau181, phosphorylated tau protein 181; p‐tau217, phosphorylated tau protein 217; SD, standard deviation.

^a^ ApoE non‐e4 allele carriers served as reference category.

### 3.5 Multiple logistic regression POCD models

We investigated models for explaining POCD in‐sample based on different POCD stages (Table 4, S5, and S6).

**TABLE 4:** Best‐fitting multiple logistic regression models for POCD stages.

Characteristics	OR	95% CI	*p*‐value	VIF
**POCD Stage 1: Model 6** (AIC = 440, BIC = 460)				
Intercept	0.37	0.29, 0.46	**<0.001**	
Aβ1‐40 (SD)	0.75	0.41, 1.31	0.325	5.7
Aβ1‐42 (SD)	1.13	0.66, 1.99	0.653	5.4
p‐tau181 (SD)	1.19	0.84, 3.11	0.640	2.0
p‐tau217 (SD)	1.59	1.04, 2.36	**0.026**	2.0
**POCD Stage 2: Model 4** (AIC = 281, BIC = 313)				
Intercept	0.14	0.01, 2.72	0.193	
Age	1.03	0.99, 1.07	0.210	1.1
Female sex	0.30	0.10, 0.77	**0.022**	1.1
CABG	0.18	0.04, 0.63	**0.013**	4.4
Valve surgery	0.33	0.08, 1.08	0.090	4.1
Other surgery	1.31	0.60, 2.76	0.487	1.1
POD	0.97	0.42, 2.10	0.948	1.1
AT^217^term (SD)	2.05	1.49, 2.88	**<0.001**	1.1
**POCD Stage 3: Model 4** (AIC = 196, BIC = 228)				
Intercept	0.02	0.00, 0.86	**0.049**	
Age	1.06	1.01, 1.11	**0.032**	1.1
Female sex	0.30	0.06, 0.96	0.070	1.1
CABG	0.09	0.01, 0.57	**0.026**	7.2
Valve surgery	0.14	0.01, 0.86	0.064	6.7
Other surgery	1.01	0.34, 2.63	0.990	1.1
POD	2.02	0.81, 4.77	0.115	1.0
AT^217^term (SD)	1.60	1.04, 2.35	**0.021**	1.1

Abbreviations: Aβ1‐40, amyloid beta 1‐40; Aβ1‐42, amyloid beta 1‐42; AIC, Akaike information criterion; AT^217^term, Aβ40/Aβ42*p‐tau181; BBM, blood biomarker; BIC, Bayesian information criterion; CABG, coronary bypass graft; CI, confidence interval; POD, postoperative delirium; p‐tau181, phosphorylated tau protein 181; p‐tau217, phosphorylated tau protein 217; SD, standard deviation; VIF, variance inflation factor.

#### 3.5.1 POCD Stage 1

We created various models for investigating POCD Stage 1, by considering clinical variables and BBMs (Model 1), clinical and individual hybrid ratios such as the Aβ1‐42/1‐40 (Model 2), AT^181^term (Model 3) or AT^217^term (Model 4), only clinical parameters (Model 5), and exclusively BBMs (Model 6) (Tables S5 and S6). The best model was Model 5 for POCD Stage 1. P‐tau217 is significantly associated with POCD Stage 1, with an OR of 1.59 (95% CI 1.04–2.36, *p* = 0.026, Table 4). If clinical parameters are included, Model 4 proved to be the best‐fitting model, showing an association between AT^217^term and POCD Stage 1^,^ with an OR of 1.61 (95% CI 1.21–2.22, *p* = 0.002, Table 4, Table S5).

#### 3.5.2 POCD Stage 2

Model 4 appeared to be the best‐fitting model for POCD Stage 2. In Model 4, the AT^217^term with an OR of 2.05 (95% CI 1.49–2.88, Table 4) was significantly associated with POCD Stage 2. Female sex and CABG were less strongly associated with POCD Stage 2 (Table 4).

#### 3.5.3 POCD Stage 3

Model 4 with the AT^217^term proved to be the best‐fitting model for POCD Stage 3 (OR = 1.60, 95% CI 1.04–2.35, *p* = 0.021, Table 4). Significant clinical variables for POCD Stage 3 in Model 4 are age (OR = 1.06, 95% CI 1.01–1.11, *p* = 0.032) and CABG (OR = 0.09, 95% CI 0.01–0.57, *p* = 0.026, Table 4). Overall, the AT^217^term is a significant in‐sample predictor over all POCD stages with low collinearity (VIF in Table 4). The ApoE4 geno‐ and proteotype were not associated with any stage of POCD in our sample when included in regression Models 1, 4, 5, and 6 (Table S6).

#### 3.5.4 Baseline adjustment for POCD at 12 months

In addition to this approach, we also conducted an analysis in which we adjusted the 12‐month MoCA score for baseline. Model 6 emerged as the most appropriate model, with the lowest AIC and BIC values. In this analysis, p‐tau217 emerged as the biomarker associated with a significantly higher risk of POCD (β −0.83, 95% CI 0.39 to −1.3, *p* < 0.0001, Table S4).

### 3.6 Relationship between POD and POCD via marginal effect approach

The estimated risk of POCD Stage 1 is 24.81% if all patients experienced POD, and 27.66% if none experienced POD. This corresponds to a risk ratio (RR) of 0.90, suggesting that the presence of POD is associated with a slightly lower risk of POCD Stage 1. The estimated risk of POCD Stage 2 amounts to 13.58% if all patients experienced POD, and 13.77% if none experienced POD. An RR of 0.99 indicates virtually no association between POD and the risk of POCD Stage 2. The estimated risk of POCD Stage 3 is 12.88% if all patients experienced POD, and 6.54% if none experienced POD. An RR of 1.97 suggests that the presence of POD is associated with a higher risk of POCD Stage 3.

## 4 DISCUSSION

The key findings from the regression analysis demonstrate an independent association between early BBMs of AD pathology (AT^217^term, but none of the other BBMs investigated) and a higher risk for different in‐sample stages of our primary outcome POCD after cardiac surgery as the primary exposure. Although p‐tau217 levels were significantly higher in patients with POCD than those without POCD, p‐tau217 alone was not independently associated with POCD Stages 2 and 3 in the best selected multivariable models. Aβ1‐42/1‐40 ratio and p‐tau217 may identify individuals with subclinical neurodegeneration who are more vulnerable to POCD. Evidence suggests that these biomarkers become abnormal earlier in the AD pathological cascade than does p‐tau181,^29^, ^30^ which may explain why AT^217^term emerged as a significant in‐sample predictor of POCD. This supports the hypothesis that patients with subclinical AD pathology are more vulnerable to POCD, and that BBMs could help identify them preoperatively for preventive strategies. Age is a well‐established risk factor for POCD, and its consistent significance across multiple logistic regression models in our study is therefore not unexpected. The observation of lower preoperative plasma Aβ peptide levels in patients who developed POCD extend the published findings.^10^, ^31^ In one trial, preoperative Aβ40 and Aβ42 levels in blood plasma were significantly reduced in patients with POCD, 3 months after CABG, and the Aβ42/40 ratio was not predictive of POCD.^10^ In another trial of individuals undergoing total hip replacement surgery, CSF levels of p‐tau181/Aβ1‐42 were significantly higher and Aβ1‐42 levels were significantly lower in individuals with POCD, 7 days after surgery.^31^ However, both studies employed different instruments for cognitive assessment than the present study. In addition, the latter trial assessed CSF rather than BBMs, reported short‐term cognitive impairment only,^31^ and did not examine the Aβ1‐42/1‐40 ratio, which would have enabled a stronger diagnostic contrast for brain β‐amyloidosis than Aβ1‐42 alone.^32^, ^33^ Furthermore, follow‐up was limited to a relatively short postoperative interval, which may have restricted the detection of longer‐term cognitive trajectories or delayed manifestations of neurodegenerative vulnerability. Thus, the novelty of our study lies in investigating POCD over a longer period and the use of relevant BBMs to capture early AD. Specifically, we evaluated p‐tau217, the Aβ1‐42/Aβ1‐40 ratio, and the composite AT^217^term, providing a comprehensive window into the early stages of AD‐related pathology. Of note, we demonstrate for the first time via BBM analyses that an AD‐related biomarker signature plays a key role in POCD at 12 months after cardiac surgery. Elevated plasma p‐tau217 levels and the AT^217^term in patients who developed POCD compared to those who remained cognitively stable reflect an underlying neurodegenerative vulnerability unmasked by surgical stress. Notably, the AT^217^term retained significance in multivariable models, highlighting its robustness and potential utility as a preoperative risk predictor in our exploratory analysis. These findings support the emerging paradigm that POCD may not be a purely postoperative phenomenon, but rather a clinical manifestation of pre‐existing cognitive fragility in individuals with AD pathology. The integration of BBMs (p‐tau217 and AT^217^term) into the preoperative risk assessment could enable the early identification of high‐risk patients, paving the way for personalized monitoring, preventive interventions, and improved long‐term outcomes in vulnerable populations undergoing cardiac surgery. It is also conceivable that within an AD pathology, both POD and BBMs have an influence on the development of POCD. However, our univariate analysis showed that this applies only to advanced POCD Stage 3, but not POCD Stages 1 and 2. POD may therefore be a contributing factor in the case of a cognitive impairment more severe than POCD. This is also consistent with findings revealing Aβ pathology and axonal neurodegeneration in POD,^34^, ^35^ suggesting that ongoing neurodegeneration in combination with POD may also affect the development of the more advanced POCD Stage 3. Although we found POD to be a minor factor and only a POCD Stage 3 predictor, we can assume from our marginal effect approach that such POD is associated with a higher risk for POCD Stage 3. POD may well mediate an association between early AD pathology and POCD at the latter's advanced Stage 3, especially when early AD pathology (AT^217^term) is already present.

### 4.1 Limitations

Limitations include first the inherent clinical heterogeneity of patients undergoing cardiac surgery, including differences in surgical procedures, anesthesia protocols, and the presence of comorbidities. Second, cerebral β‐amyloidosis was not confirmed using CSF biomarkers as the gold standard for neuropathological validation. However, recent studies have demonstrated that the combination of plasma p‐tau217 and the Aβ42/Aβ40 ratio achieves sensitivities and specificities exceeding 90% for detecting cerebral β‐amyloidosis,^36^ suggesting that this BBM profile can reliably capture an AD‐related trait and may reduce the need for CSF confirmation in research settings. Third, cognitive function was assessed using the MoCA, a widely validated screening tool that efficiently captures core cognitive domains. Although the MoCA lacks the granularity of a comprehensive neuropsychological battery, it is well‐suited for large‐scale clinical studies and has demonstrated strong sensitivity to postoperative cognitive changes. Its use enhances the feasibility and reproducibility of cognitive assessments in real‐world surgical populations.^37^, ^38^ Finally, the predictive performance of the BBMs was evaluated within the study cohort (in‐sample), and thus external validation in an independent cohort is essential to confirm the generalizability and robustness of the findings.

## 5 CONCLUSIONS

Our exploratory findings potentially have important clinical implications, demonstrating that AD‐related pathology, as reflected by the BBM p‐tau217 and the AT^217^term, can be identified prior to scheduled cardiac surgery. This study provides a critical but so far exploratory foundation for reconsidering perioperative risk assessments in older adults, particularly with respect to their cognitive vulnerability. The presence of an AD‐BBM profile may support individualized risk–benefit assessments in exploratory analysis, may inform shared decision‐making, and may guide the urgency of surgical procedures in future assessments. However, we state firmly that no causal relationship between BBMs and POCD has been proven; however, multiple regression models have demonstrated a significant association between the AT^217^term blood biomarker and a higher risk for POCD as our study's primary outcome. Provided that our study results are validated externally, integrating the BBMs into routine preoperative screening could contribute to more personalized perioperative management strategies aimed at minimizing POCD.

## AUTHOR CONTRIBUTIONS

Niels Hansen, Monika Sadlonova, Christine A. F. von Arnim, Jens Wiltfang wrote the manuscript. All other authors have revised the manuscript for important inteletual content.

## CONFLICT OF INTEREST STATEMENT

C.A.F.vA. has served on advisory boards for Biogen, Roche, Novo Nordisk, Biontech, Lilly, Dr. Willmar Schwabe GmbH (Gesellschaft mit beschränkter Haftung) &Co. KG, RoX Health GmbH, Bosch Health Campus, and MindAhead UG (Unternehmergesellschaft). She has also received honoraria from Lilly, Novo Nordisk, Roche, Novartis, Medical Tribune Verlagsgesellschaft mbH, Landesvereinigung für Gesundheit und Akademie für Sozialmedizin Niedersachsen e. V. (eingetragener Verein), FomF (Forum für medizinische Fortbildung) GmbH, and Dr. Willmar Schwabe GmbH &Co. KG; as well as research funding from the Innovationsfond (Fund of the Federal Joint Committee, Gemeinsamer Bundesausschuss, G‐BA (Gemeinsamer Bundesausschuss) Grants No. VF1_2016‐201; 01NVF21010; 01VSF21019). She further received funding from the DFG (GRK 2824: RP9; 540861493). C.M.C. received funding from the National Heart, Lung, and Blood Institute (grant R01HL155301) and stipends from Elsevier for editorial work for General Hospital Psychiatry. J.W. has received honoraria for serving on scientific advisory boards for Abbot, Biogen, Boehringer‐Ingelheim, Eli Lilly, F. Hoffmann‐La Roche, Immunogenetics, and MSD SHARP & DOHME, and has also received honoraria for lectures sponsored by Eli Lilly, Pfizer, Janssen, MSD SHARP & DOHME, Amgen, Roche Pharma, Actelion Pharmaceuticals, Guangzhou Glorylen Medical Technology Co. (China), and Beijing Yibai Science and Technology Ltd. N.H. received funding from the DFG (Deutsche Forschungsgemeinschaft) (530229798) and honoraria for traveling and lecture from Eisai GmbH and Eli Lilly. Furthermore, he has received travel honoraria from Bristol Myers Squibb. All other authors report no competing interests. Author disclosures are available in the Supporting Information.

## CONSENT STATEMENT

In this study all human subjects provided written informed consent.

## Supporting information



Supporting information

Supporting information

Supporting information

Supporting information

Supporting information

Supporting information

Supporting information

Supporting Information
