# Supplementary material for: Early Alzheimer´s disease blood biomarkers are associated with a higher risk for postoperative long‐term cognitive decline: Insights from the FINDERI study
Source: Alzheimers Dement. 2026 Jul 14;22(7):e71631. doi: 10.1002/alz.71631 (PMC13368704; doi:10.1002/alz.71631)
Supplement: Supplementary file 1 — Supporting information [file ALZ-22-e71631-s004.docx]

**Supplement Table 1: ApoE proteotype stratified by ApoE genotype** **and different POCD stages**

|  | **Complete cohort** | **With POCD-Status** | **POCD Stage 1** | | | **POCD Stage 2** | | | **POCD Stage 3** | | |
| --- | --- | --- | --- | --- | --- | --- | --- | --- | --- | --- | --- |
| **Characteristic** | **N = 504**^1^ | **N = 394**^1^ | **No** **POCD** N = 289^1^ | **POCD** N = 105^1^ | **p-value**^2^ | **No** **POCD** N = 342^1^ | **POCD** N = 52^1^ | **p-value**^2^ | **No** **POCD** N = 364^1^ | **POCD** N = 30^1^ | **p-value**^2^ |
| **ApoE non ApoE4 (µg/ml)** | 21.74 ± 13.22 | 21.49 ± 13.16 | 20.51 ± 12.03 | 24.26 ± 15.67 | 0.070 | 21.07 ± 12.73 | 24.54 ± 15.81 | 0.234 | 21.26 ± 12.82 | 25.14 ± 17.83 | 0.405 |
| N | 344 | 270 | 199 | 71 |  | 237 | 33 |  | 254 | 16 |  |
| **ApoE in heterozygous ApoE4 (µg/ml)** | 13.73 ± 6.10 | 13.84 ± 6.14 | 13.62 ± 6.32 | 14.36 ± 5.76 | 0.573 | 13.69 ± 6.24 | 14.48 ± 5.78 | 0.613 | 13.65 ± 6.13 | 15.08 ± 6.31 | 0.457 |
| N | 123 | 98 | 69 | 29 |  | 80 | 18 |  | 85 | 13 |  |
| **ApoE in homozygous ApoE4 (µg/ml)** | 22.43 ± 28.21 | 23.50 ± 31.03 | 25.23 ± 32.40 | 7.90 ± NA |  | 23.50 ± 31.03 | NA ± NA |  | 23.50 ± 31.03 | NA ± NA |  |
| N | 12 | 10 | 9 | 1 |  | 10 | 0 |  | 10 | 0 |  |
| **ApoE4 non ApoE4 (µg/ml)** | 0.05 ± 0.47 | 0.06 ± 0.53 | 0.03 ± 0.17 | 0.14 ± 1.00 | 0.341 | 0.06 ± 0.57 | 0.03 ± 0.05 | 0.367 | 0.06 ± 0.55 | 0.03 ± 0.05 | 0.451 |
| N | 344 | 270 | 199 | 71 |  | 237 | 33 |  | 254 | 16 |  |
| **ApoE4 in heterozygous ApoE4 (µg/ml)** | 6.60 ± 4.12 | 6.77 ± 4.17 | 6.63 ± 3.81 | 7.09 ± 4.98 | 0.659 | 6.90 ± 4.36 | 6.20 ± 3.24 | 0.446 | 6.76 ± 4.27 | 6.82 ± 3.57 | 0.963 |
| N | 123 | 98 | 69 | 29 |  | 80 | 18 |  | 85 | 13 |  |
| **ApoE4 in homozygous ApoE4 (µg/ml)** | 31.97 ± 27.03 | 32.13 ± 29.83 | 33.80 ± 31.14 | 17.10 ± NA |  | 32.13 ± 29.83 | NA ± NA |  | 32.13 ± 29.83 | NA ± NA |  |
| N | 12 | 10 | 9 | 1 |  | 10 | 0 |  | 10 | 0 |  |
| **Ratio ApoE4/ApoE non ApoE4** | 0.00 ± 0.02 | 0.00 ± 0.03 | 0.00 ± 0.03 | 0.00 ± 0.03 | 0.708 | 0.00 ± 0.03 | 0.00 ± 0.00 | 0.161 | 0.00 ± 0.03 | 0.00 ± 0.00 | 0.161 |
| N | 344 | 270 | 199 | 71 |  | 237 | 33 |  | 254 | 16 |  |
| **Ratio ApoE4/ApoE in heterozygous ApoE4** | 0.47 ± 0.17 | 0.48 ± 0.16 | 0.48 ± 0.16 | 0.48 ± 0.17 | 0.838 | 0.49 ± 0.17 | 0.43 ± 0.13 | 0.102 | 0.49 ± 0.16 | 0.45 ± 0.14 | 0.437 |
| N | 123 | 98 | 69 | 29 |  | 80 | 18 |  | 85 | 13 |  |
| **Ratio ApoE4/ApoE in homozygous ApoE4** | 1.97 ± 0.61 | 1.99 ± 0.67 | 1.97 ± 0.70 | 2.16 ± NA |  | 1.99 ± 0.67 | NA ± NA |  | 1.99 ± 0.67 | NA ± NA |  |
| N | 12 | 10 | 9 | 1 |  | 10 | 0 |  | 10 | 0 |  |
| ^1^Mean ± SD | | | | | | | | | | | |
| ^2^Welch Two Sample t-test | | | | | | | | | | | |

Abbreviations: ApoE4 = ApoliproteinE4, ApoE = Apoliprotein E, N = number, POCD = postoperative cognitive dysfunction
