# Supplementary material for: Early Alzheimer´s disease blood biomarkers are associated with a higher risk for postoperative long‐term cognitive decline: Insights from the FINDERI study
Source: Alzheimers Dement. 2026 Jul 14;22(7):e71631. doi: 10.1002/alz.71631 (PMC13368704; doi:10.1002/alz.71631)
Supplement: Supplementary file 2 — Supporting information [file ALZ-22-e71631-s007.docx]

Supplement Table 2: Area under the curves and optimal cutoffs for 12-months POCD

| Variable | AUC (95% CI) | p-value | Optimal cut-off | Sensitivity (95% CI) | Specificity (95% CI) | PPV (95% CI) | NPV (95% CI) |
| --- | --- | --- | --- | --- | --- | --- | --- |
| **POCD: Stage 1** |  |  |  |  |  |  |  |
| Aβ1-40 | 0.5318 (0.4638, 0.5987) | 0.369 | >360.73 | 0.5392 (0.4377, 0.6384) | 0.5464 (0.4861, 0.6058) | 0.3022 (0.2365, 0.3745) | 0.765 (0.7, 0.8219) |
| Aβ1-42 | 0.5311 (0.4614, 0.5988) | 0.372 | >28.97 | 0.5392 (0.4377, 0.6384) | 0.5393 (0.479, 0.5988) | 0.2989 (0.2338, 0.3706) | 0.7626 (0.6971, 0.8201) |
| p-tau181/p-tau217 ratio | 0.5032 (0.4348, 0.5695) | 0.943 | >0.4149 | 0.5098 (0.4089, 0.6101) | 0.5214 (0.4612, 0.5812) | 0.2796 (0.2164, 0.3499) | 0.7449 (0.6779, 0.8043) |
| ApoE | 0.5515 (0.4843, 0.6175) | 0.133 | <16.2 | 0.5294 (0.428, 0.629) | 0.5233 (0.4629, 0.5832) | 0.2888 (0.225, 0.3594) | 0.7526 (0.6857, 0.8116) |
| ApoE4 allele count | 0.5048 (0.4559, 0.5599) | 0.843 | <1 | 0.297 (0.2012, 0.3961) | 0.7194 (0.6627, 0.7714) | 0.2778 (0.1959, 0.3722) | 0.738 (0.6814, 0.7893) |
| **POCD: Stage 2** |  |  |  |  |  |  |  |
| Aβ1-40 | 0.5536 (0.4662, 0.6367) | 0.234 | <371.36 | 0.5294 (0.3846, 0.6707) | 0.5468 (0.4915, 0.6013) | 0.1525 (0.103, 0.2141) | 0.8829 (0.8308, 0.9235) |
| Aβ1-42 | 0.5215 (0.435, 0.6081) | 0.620 | <29.62 | 0.5098 (0.366, 0.6525) | 0.5045 (0.4493, 0.5597) | 0.1368 (0.0914, 0.194) | 0.8698 (0.8138, 0.9139) |
| p-tau181/p-tau217 ratio | 0.5574 (0.467, 0.6462) | 0.223 | >0.4065 | 0.549 (0.4034, 0.6887) | 0.5438 (0.4885, 0.5984) | 0.1564 (0.1065, 0.2181) | 0.8867 (0.8349, 0.9268) |
| ApoE | 0.5295 (0.4379, 0.6183) | 0.503 | <16.2 | 0.5294 (0.428, 0.629) | 0.5233 (0.4629, 0.5832) | 0.2888 (0.225, 0.3594) | 0.7526 (0.6857, 0.8116) |
| ApoE4 allele count | 0.5339 (0.4671, 0.6105) | 0.327 | <1 | 0.3529 (0.2243, 0.4493) | 0.7256 (0.6739, 0.7732) | 0.1667 (0.1019, 0.2506) | 0.8782 (0.8333, 0.9147) |
| **POCD: Stage 3** |  |  |  |  |  |  |  |
| Aβ1-40 | 0.6039 (0.4942, 0.705) | 0.062 | <378.55 | 0.5862 (0.3894, 0.7648) | 0.5864 (0.5331, 0.6383) | 0.1043 (0.0619, 0.1617) | 0.9452 (0.9062, 0.9714) |
| Aβ1-42 | 0.5444 (0.4276, 0.6564) | 0.462 | <29.95 | 0.5517 (0.3569, 0.7355) | 0.5354 (0.4819, 0.5884) | 0.0889 (0.0517, 0.1403) | 0.9356 (0.8925, 0.9653) |
| p-tau181/p-tau217 ratio | 0.6035 (0.4839, 0.7128) | 0.092 | >0.4029 | 0.5517 (0.3569, 0.7355) | 0.5524 (0.4989, 0.6051) | 0.092 (0.0535, 0.145) | 0.9375 (0.8955, 0.9663) |
| ApoE | 0.5197 (0.4052, 0.6309) | 0.725 | <16.2 | 0.5294 (0.428, 0.629) | 0.5233 (0.4629, 0.5832) | 0.2888 (0.225, 0.3594) | 0.7526 (0.6857, 0.8116) |
| ApoE4 allele count | 0.5820 (0.4970, 0.6878) | 0.064 | <1 | 0.4483 (0.2645, 0.6431) | 0.7286 (0.6787, 0.7745) | 0.1204 (0.0657, 0.197) | 0.941 (0.9059, 0.9659) |
| < values below cutoff are classified as healthy; > values above cutoff are classified as healthy; CI = confidence interval (exact binomial confidence limits)  Abbreviations: Aβ1-42= amyloid-β1-42, Aβ1-40 = amyloid-β1-40, ApoE4 = Apoliprotein E4, ApoE = Apoliprotein E, AT^181^term = amyloid-β1-40/ 1-42*p-tau181, AT^217^term= amyloid-β1-40/1-42*p-tau217, AUC = area under the curve with logit-transformed, permutation based 95% confidence interval, NPV = negative predictive value, POCD = postoperative cognitive dysfunction, PPV = positive predictive value, p-tau181 = phosphorylated tau protein 181, p-tau217 = phosphorylated tau protein 217. | | | | | | | |
