# Supplementary material for: Early Alzheimer´s disease blood biomarkers are associated with a higher risk for postoperative long‐term cognitive decline: Insights from the FINDERI study
Source: Alzheimers Dement. 2026 Jul 14;22(7):e71631. doi: 10.1002/alz.71631 (PMC13368704; doi:10.1002/alz.71631)
Supplement: Supplementary file 3 — Supporting information [file ALZ-22-e71631-s008.docx]

Supplement Table 3: Univariate logistic regressions in different stages of POCD

|  | **POCD Stage 1** | | | | **POCD Stage 2** | | | | **POCD Stage 3** | | | |
| --- | --- | --- | --- | --- | --- | --- | --- | --- | --- | --- | --- | --- |
| **Characteristic** | **N** | **OR** | **95% CI** | **p-value** | **N** | **OR** | **95% CI** | **p-value** | **N** | **OR** | **95% CI** | **p-value** |
| **BMI** | 390 | 1.01 | 0.96, 1.06 | 0.740 | 390 | 1.03 | 0.97, 1.09 | 0.351 | 390 | 0.99 | 0.91, 1.07 | 0.795 |
| **Renal failure** | 394 | 0.80 | 0.37, 1.58 | 0.533 | 394 | 1.37 | 0.57, 2.99 | 0.450 | 394 | 1.12 | 0.32, 3.04 | 0.841 |
| **CABG** | 394 | 0.67 | 0.42, 1.06 | 0.087 | 394 | 0.58 | 0.32, 1.06 | 0.073 | 394 | 0.63 | 0.30, 1.36 | 0.226 |
| **Valve surgery** | 394 | 1.14 | 0.72, 1.78 | 0.577 | 394 | 1.24 | 0.69, 2.23 | 0.466 | 394 | 1.00 | 0.46, 2.11 | 0.994 |
| **Other surgery** | 394 | 1.43 | 0.83, 2.42 | 0.186 | 394 | 1.73 | 0.87, 3.28 | 0.103 | 394 | 1.47 | 0.60, 3.33 | 0.370 |
| **ApoE4/ApoE proteotype (SD)** | 381 | 0.94 | 0.73, 1.17 | 0.611 | 381 | 0.91 | 0.62, 1.21 | 0.584 | 381 | 1.06 | 0.70, 1.41 | 0.742 |
| **ApoE (SD)** | 381 | 1.17 | 0.94, 1.45 | 0.139 | 381 | 1.12 | 0.84, 1.44 | 0.384 | 381 | 1.09 | 0.73, 1.48 | 0.631 |
| **ApoE4 (SD)** | 381 | 0.94 | 0.69, 1.17 | 0.628 | 381 | 0.92 | 0.57, 1.21 | 0.659 | 381 | 1.05 | 0.68, 1.34 | 0.754 |
| Abbreviations: ApoE4 = Apolipoprotein E4, ApoE = Apolipoprotein E, BMI = body mass index, CABG = coronary bypass graft, CI = Confidence Interval, N = number, OR = Odds Ratio, POD = postoperative delirium, SD = standard deviation | | | | | | | | | | | | |
