# Supplementary material for: Early Alzheimer´s disease blood biomarkers are associated with a higher risk for postoperative long‐term cognitive decline: Insights from the FINDERI study
Source: Alzheimers Dement. 2026 Jul 14;22(7):e71631. doi: 10.1002/alz.71631 (PMC13368704; doi:10.1002/alz.71631)
Supplement: Supplementary file 4 — Supporting information [file ALZ-22-e71631-s002.docx]

Supplement Table 4:

1. Univariate linear regressions for 12mo-MoCA.

| **Characteristic** | **N** | **β** | **95% CI** | **p-value** |
| --- | --- | --- | --- | --- |
| **Baseline MoCA (0-30)** | 394 | 0.65 | 0.57, 0.73 | **<0.001** |
| **Aβ40, SD** | 382 | -0.37 | -0.72, -0.03 | **0.034** |
| **Aβ42, SD** | 382 | -0.26 | -0.61, 0.09 | 0.142 |
| **Aβ42/Aβ40 ratio, SD** | 382 | 0.39 | 0.02, 0.75 | **0.039** |
| **pTau181, SD** | 388 | -0.33 | -0.64, -0.02 | **0.037** |
| **AT181-Term, SD** | 382 | -0.34 | -0.66, -0.03 | **0.034** |
| **pTau217, SD** | 382 | -1.0 | -1.4, -0.62 | **<0.001** |
| **AT217-Term, SD** | 382 | -1.1 | -1.5, -0.67 | **<0.001** |
| **pTau181/pTau217 ratio, SD** | 382 | 0.15 | -0.20, 0.51 | 0.392 |
| **ApoE4 e4-allele carrier** | 379 | -0.25 | -1.0, 0.52 | 0.525 |
| **ApoE4/ApoE, SD** | 381 | -0.04 | -0.37, 0.30 | 0.823 |
| **ApoE, SD** | 381 | -0.03 | -0.38, 0.31 | 0.845 |
| **ApoE4, SD** | 381 | 0.13 | -0.20, 0.46 | 0.441 |
| **Age** | 394 | -0.09 | -0.13, -0.05 | **<0.001** |
| **BMI** | 390 | -0.05 | -0.12, 0.03 | 0.198 |
| **Renal failure** | 394 | -0.16 | -1.2, 0.89 | 0.765 |
| **Female sex** | 394 | 0.94 | 0.09, 1.8 | **0.030** |
| **CABG** | 394 | 0.28 | -0.45, 1.0 | 0.452 |
| **Valve surgery** | 394 | -0.21 | -0.90, 0.48 | 0.548 |
| **Other Surgery** | 394 | -0.19 | -1.0, 0.66 | 0.660 |
| **Surgery duration (h)** | 394 | 0.23 | -0.02, 0.48 | 0.077 |
| **POD** | 388 | -1.7 | -2.6, -0.85 | **<0.001** |
| Abbreviation: CI = Confidence Interval | | | | |

1. Multiple linear regression models for 12 months MoCA adjusted for baseline MoCA

|  | 1 - Clinical + BBM | | | | 2 - Clinical + Aβ42/40 | | | | 3 - Clinical + AT181 | | | | 4 - Clinical + AT217 | | | | 5 - Clinical only | | | | 6 - BBM only | | | |
| --- | --- | --- | --- | --- | --- | --- | --- | --- | --- | --- | --- | --- | --- | --- | --- | --- | --- | --- | --- | --- | --- | --- | --- | --- |
| **Characteristic** | **β** | **95% CI** | **p-value** | **VIF** | **β** | **95% CI** | **p-value** | **VIF** | **β** | **95% CI** | **p-value** | **VIF** | **β** | **95% CI** | **p-value** | **VIF** | **β** | **95% CI** | **p-value** | **VIF** | **β** | **95% CI** | **p-value** | **VIF** |
| **(Intercept)** | 9.8 | 6.0, 14 | **<0.001** |  | 10 | 6.6, 14 | **<0.001** |  | 10 | 6.5, 14 | **<0.001** |  | 9.7 | 6.0, 13 | **<0.001** |  | 10 | 6.4, 14 | **<0.001** |  | 9.6 | 7.5, 12 | **<0.001** |  |
| **Baseline MoCA (0-30)** | 0.61 | 0.52, 0.70 | **<0.001** | 1.2 | 0.62 | 0.53, 0.71 | **<0.001** | 1.2 | 0.61 | 0.52, 0.71 | **<0.001** | 1.2 | 0.61 | 0.52, 0.69 | **<0.001** | 1.2 | 0.63 | 0.54, 0.71 | **<0.001** | 1.2 | 0.63 | 0.55, 0.71 | **<0.001** | 1.0 |
| **Female sex** | 0.93 | 0.24, 1.6 | **0.009** | 1.1 | 0.94 | 0.23, 1.7 | **0.009** | 1.1 | 0.92 | 0.22, 1.6 | **0.010** | 1.1 | 0.90 | 0.20, 1.6 | **0.011** | 1.1 | 0.87 | 0.19, 1.6 | **0.013** | 1.1 |  |  |  |  |
| **Age** | -0.01 | -0.04, 0.03 | 0.727 | 1.2 | -0.02 | -0.05, 0.02 | 0.376 | 1.2 | -0.01 | -0.05, 0.02 | 0.412 | 1.1 | 0.00 | -0.04, 0.03 | 0.809 | 1.2 | -0.01 | -0.05, 0.02 | 0.460 | 1.1 |  |  |  |  |
| **CABG** | 0.69 | -0.24, 1.6 | 0.145 | 2.7 | 0.61 | -0.33, 1.5 | 0.204 | 2.6 | 0.69 | -0.25, 1.6 | 0.148 | 2.7 | 0.70 | -0.22, 1.6 | 0.137 | 2.6 | 0.53 | -0.37, 1.4 | 0.247 | 2.5 |  |  |  |  |
| **Valve surgery** | 0.36 | -0.50, 1.2 | 0.412 | 2.5 | 0.31 | -0.56, 1.2 | 0.483 | 2.5 | 0.39 | -0.48, 1.3 | 0.382 | 2.5 | 0.39 | -0.47, 1.2 | 0.373 | 2.5 | 0.20 | -0.63, 1.0 | 0.630 | 2.4 |  |  |  |  |
| **Other Surgery** | -0.04 | -0.74, 0.67 | 0.912 | 1.1 | -0.18 | -0.89, 0.53 | 0.616 | 1.1 | -0.19 | -0.90, 0.52 | 0.598 | 1.1 | -0.12 | -0.82, 0.58 | 0.739 | 1.1 | -0.15 | -0.86, 0.55 | 0.668 | 1.1 |  |  |  |  |
| **POD** | -0.36 | -1.1, 0.36 | 0.327 | 1.1 | -0.43 | -1.2, 0.30 | 0.246 | 1.1 | -0.42 | -1.1, 0.30 | 0.251 | 1.1 | -0.32 | -1.0, 0.40 | 0.383 | 1.1 | -0.45 | -1.2, 0.26 | 0.213 | 1.1 |  |  |  |  |
| **Aβ40, SD** | 0.30 | -0.36, 0.96 | 0.371 | 6.0 |  |  |  |  |  |  |  |  |  |  |  |  |  |  |  |  | 0.18 | -0.46, 0.83 | 0.575 | 5.7 |
| **Aβ42, SD** | -0.04 | -0.68, 0.60 | 0.903 | 5.7 |  |  |  |  |  |  |  |  |  |  |  |  |  |  |  |  | 0.06 | -0.56, 0.69 | 0.839 | 5.4 |
| **pTau181, SD** | 0.08 | -0.21, 0.38 | 0.583 | 1.5 |  |  |  |  |  |  |  |  |  |  |  |  |  |  |  |  | 0.10 | -0.19, 0.39 | 0.497 | 1.4 |
| **pTau217, SD** | -0.81 | -1.2, -0.38 | **<0.001** | 1.8 |  |  |  |  |  |  |  |  |  |  |  |  |  |  |  |  | -0.83 | -1.3, -0.40 | **<0.001** | 1.7 |
| **Aβ42/Aβ40 ratio, SD** |  |  |  |  | -0.03 | -0.33, 0.27 | 0.848 | 1.1 |  |  |  |  |  |  |  |  |  |  |  |  |  |  |  |  |
| **AT181-Term, SD** |  |  |  |  |  |  |  |  | -0.18 | -0.44, 0.07 | 0.155 | 1.0 |  |  |  |  |  |  |  |  |  |  |  |  |
| **AT217-Term, SD** |  |  |  |  |  |  |  |  |  |  |  |  | -0.64 | -1.0, -0.27 | **<0.001** | 1.1 |  |  |  |  |  |  |  |  |
| R² | 0.420 |  |  |  | 0.395 |  |  |  | 0.398 |  |  |  | 0.414 |  |  |  | 0.408 |  |  |  | 0.406 |  |  |  |
| Adjusted R² | 0.403 |  |  |  | 0.382 |  |  |  | 0.385 |  |  |  | 0.401 |  |  |  | 0.397 |  |  |  | 0.398 |  |  |  |
| AIC | 1,846 |  |  |  | 1,856 |  |  |  | 1,854 |  |  |  | 1,844 |  |  |  | 1,880 |  |  |  | 1,843 |  |  |  |
| BIC | 1,897 |  |  |  | 1,895 |  |  |  | 1,893 |  |  |  | 1,883 |  |  |  | 1,915 |  |  |  | 1,870 |  |  |  |
| No. Obs. | 382 |  |  |  | 382 |  |  |  | 382 |  |  |  | 382 |  |  |  | 388 |  |  |  | 382 |  |  |  |
| Abbreviations: CI = Confidence Interval, VIF = Variance Inflation Factor | | | | | | | | | | | | | | | | | | | | | | | | |
