# Supplementary material for: Early Alzheimer´s disease blood biomarkers are associated with a higher risk for postoperative long‐term cognitive decline: Insights from the FINDERI study
Source: Alzheimers Dement. 2026 Jul 14;22(7):e71631. doi: 10.1002/alz.71631 (PMC13368704; doi:10.1002/alz.71631)
Supplement: Supplementary file 5 — Supporting information [file ALZ-22-e71631-s006.docx]

Table 5: Multiple logistic regression for POCD

**Multiple logistic regression for 12 months-POCD Stage 1**

| **Models** | **1 - Clinical + BBM** | | | | **2 - Clinical + Aβ42/40** | | | | **3 – Clinical + AT^181^term** | | | | **4 – Clinical + AT^217^term** | | | | **5 – Clinical only** | | | | **6 – BBM only** | | | |
| --- | --- | --- | --- | --- | --- | --- | --- | --- | --- | --- | --- | --- | --- | --- | --- | --- | --- | --- | --- | --- | --- | --- | --- | --- |
| **Characteristic** | **OR** | **95% CI** | **p-value** | **VIF** | **OR** | **95% CI** | **p-value** | **VIF** | **OR** | **95% CI** | **p-value** | **VIF** | **OR** | **95% CI** | **p-value** | **VIF** | **OR** | **95% CI** | **p-value** | **VIF** | **OR** | **95% CI** | **p-value** | **VIF** |
| **(Intercept)** | 0.66 | 0.07, 5.77 | 0.703 |  | 0.30 | 0.04, 2.37 | 0.255 |  | 0.60 | 0.07, 5.19 | 0.637 |  | 0.77 | 0.09, 6.65 | 0.808 |  | 0.36 | 0.05, 2.71 | 0.322 |  | 0.37 | 0.29, 0.46 | **<0.001** |  |
| **Female sex** | 0.60 | 0.31, 1.11 | 0.115 | 1.1 | 0.59 | 0.30, 1.08 | 0.097 | 1.1 | 0.63 | 0.33, 1.16 | 0.154 | 1.1 | 0.62 | 0.32, 1.14 | 0.138 | 1.0 | 0.61 | 0.32, 1.10 | 0.112 | 1.1 |  |  |  |  |
| **Age** | 1.00 | 0.97, 1.04 | 0.772 | 1.1 | 1.01 | 0.98, 1.04 | 0.371 | 1.1 | 1.01 | 0.98, 1.04 | 0.714 | 1.1 | 1.00 | 0.97, 1.03 | 0.921 | 1.1 | 1.01 | 0.98, 1.04 | 0.464 | 1.0 |  |  |  |  |
| **CABG** | 0.42 | 0.17, 0.98 | 0.055 | 3.3 | 0.49 | 0.20, 1.10 | 0.094 | 3.1 | 0.42 | 0.17, 0.98 | 0.053 | 3.3 | 0.44 | 0.18, 1.01 | 0.061 | 3.2 | 0.48 | 0.21, 1.06 | 0.078 | 2.9 |  |  |  |  |
| **Valve surgery** | 0.57 | 0.24, 1.27 | 0.193 | 3.2 | 0.66 | 0.29, 1.39 | 0.292 | 2.9 | 0.58 | 0.24, 1.27 | 0.191 | 3.1 | 0.60 | 0.25, 1.30 | 0.215 | 3.0 | 0.68 | 0.31, 1.41 | 0.320 | 2.8 |  |  |  |  |
| **Other Surgery** | 1.13 | 0.61, 2.03 | 0.697 | 1.1 | 1.22 | 0.68, 2.17 | 0.494 | 1.1 | 1.23 | 0.68, 2.19 | 0.481 | 1.1 | 1.18 | 0.64, 2.11 | 0.591 | 1.1 | 1.19 | 0.66, 2.10 | 0.558 | 1.1 |  |  |  |  |
| **POD** | 0.93 | 0.49, 1.71 | 0.823 | 1.1 | 0.99 | 0.53, 1.78 | 0.971 | 1.1 | 0.93 | 0.50, 1.68 | 0.815 | 1.0 | 0.87 | 0.46, 1.58 | 0.657 | 1.1 | 0.96 | 0.52, 1.72 | 0.904 | 1.0 |  |  |  |  |
| **Aβ1-40 (SD)** | 0.74 | 0.39, 1.33 | 0.331 | 6.3 |  |  |  |  |  |  |  |  |  |  |  |  |  |  |  |  | 0.75 | 0.41, 1.31 | 0.325 | 5.7 |
| **Aβ1-42 (SD)** | 1.14 | 0.64, 2.06 | 0.651 | 5.9 |  |  |  |  |  |  |  |  |  |  |  |  |  |  |  |  | 1.13 | 0.66, 1.99 | 0.653 | 5.4 |
| **p-tau181 (SD)** | 1.17 | 0.86, 2.98 | 0.594 | 1.8 |  |  |  |  |  |  |  |  |  |  |  |  |  |  |  |  | 1.19 | 0.84, 3.11 | 0.640 | 2.0 |
| **p-tau217 (SD)** | 1.59 | 1.03, 2.38 | **0.024** | 2.0 |  |  |  |  |  |  |  |  |  |  |  |  |  |  |  |  | 1.59 | 1.04, 2.36 | **0.026** | 2.0 |
| **Aβ1-42/1-40 (SD)** |  |  |  |  | 1.08 | 0.83, 1.40 | 0.578 | 1.1 |  |  |  |  |  |  |  |  |  |  |  |  |  |  |  |  |
| **AT^181^term (SD)** |  |  |  |  |  |  |  |  | 1.82 | 1.09, 3.52 | 0.057 | 1.1 |  |  |  |  |  |  |  |  |  |  |  |  |
| **AT^217^term (SD)** |  |  |  |  |  |  |  |  |  |  |  |  | 1.61 | 1.21, 2.22 | **0.002** | 1.1 |  |  |  |  |  |  |  |  |
| AIC | 446 |  |  |  | 453 |  |  |  | 446 |  |  |  | 443 |  |  |  | 457 |  |  |  | 440 |  |  |  |
| BIC | 489 |  |  |  | 484 |  |  |  | 477 |  |  |  | 474 |  |  |  | 484 |  |  |  | 460 |  |  |  |
| No. Obs. | 382 |  |  |  | 382 |  |  |  | 382 |  |  |  | 382 |  |  |  | 388 |  |  |  | 382 |  |  |  |
| Multiple logistic regression for 12 months-POCD Stage 2   \| **Models** \| **1 - Clinical + BBM** \| \| \| \| **2 - Clinical + Aβ42/40** \| \| \| \| **3 – Clinical + AT^181^term** \| \| \| \| **4 – Clinical + AT^217^term** \| \| \| \| **5 – Clinical only** \| \| \| \| **6 – BBM only** \| \| \| \| \| --- \| --- \| --- \| --- \| --- \| --- \| --- \| --- \| --- \| --- \| --- \| --- \| --- \| --- \| --- \| --- \| --- \| --- \| --- \| --- \| --- \| --- \| --- \| --- \| --- \| \| **Characteristic** \| **OR** \| **95% CI** \| **p-value** \| **VIF** \| **OR** \| **95% CI** \| **p-value** \| **VIF** \| **OR** \| **95% CI** \| **p-value** \| **VIF** \| **OR** \| **95% CI** \| **p-value** \| **VIF** \| **OR** \| **95% CI** \| **p-value** \| **VIF** \| **OR** \| **95% CI** \| **p-value** \| **VIF** \| \| **(Intercept)** \| 0.14 \| 0.01, 2.99 \| 0.208 \|  \| 0.04 \| 0.00, 0.65 \| **0.026** \|  \| 0.10 \| 0.00, 2.14 \| 0.141 \|  \| 0.14 \| 0.01, 2.72 \| 0.193 \|  \| 0.03 \| 0.00, 0.50 \| **0.015** \|  \| 0.15 \| 0.11, 0.20 \| **<0.001** \|  \| \| **Female sex** \| 0.29 \| 0.09, 0.76 \| **0.020** \| 1.1 \| 0.31 \| 0.10, 0.77 \| **0.021** \| 1.1 \| 0.32 \| 0.10, 0.82 \| **0.029** \| 1.1 \| 0.30 \| 0.10, 0.77 \| **0.022** \| 1.1 \| 0.28 \| 0.09, 0.70 \| **0.013** \| 1.1 \|  \|  \|  \|  \| \| **Age** \| 1.03 \| 0.99, 1.07 \| 0.157 \| 1.1 \| 1.04 \| 1.00, 1.08 \| **0.047** \| 1.1 \| 1.03 \| 0.99, 1.07 \| 0.124 \| 1.1 \| 1.03 \| 0.99, 1.07 \| 0.210 \| 1.1 \| 1.04 \| 1.00, 1.08 \| **0.031** \| 1.1 \|  \|  \|  \|  \| \| **CABG** \| 0.14 \| 0.03, 0.54 \| **0.010** \| 5.5 \| 0.25 \| 0.06, 0.79 \| **0.030** \| 4.2 \| 0.16 \| 0.03, 0.57 \| **0.010** \| 5.0 \| 0.18 \| 0.04, 0.63 \| **0.013** \| 4.4 \| 0.26 \| 0.07, 0.80 \| **0.030** \| 4.0 \|  \|  \|  \|  \| \| **Valve surgery** \| 0.25 \| 0.05, 0.93 \| 0.060 \| 5.2 \| 0.44 \| 0.11, 1.34 \| 0.187 \| 4.0 \| 0.28 \| 0.06, 0.97 \| 0.069 \| 4.7 \| 0.33 \| 0.08, 1.08 \| 0.090 \| 4.1 \| 0.46 \| 0.13, 1.35 \| 0.194 \| 3.7 \|  \|  \|  \|  \| \| **Other Surgery** \| 1.25 \| 0.56, 2.66 \| 0.577 \| 1.1 \| 1.45 \| 0.68, 2.95 \| 0.319 \| 1.1 \| 1.45 \| 0.67, 3.00 \| 0.327 \| 1.1 \| 1.31 \| 0.60, 2.76 \| 0.487 \| 1.1 \| 1.42 \| 0.67, 2.88 \| 0.348 \| 1.1 \|  \|  \|  \|  \| \| **POD** \| 1.06 \| 0.45, 2.31 \| 0.887 \| 1.1 \| 1.11 \| 0.50, 2.31 \| 0.796 \| 1.1 \| 1.08 \| 0.48, 2.27 \| 0.853 \| 1.0 \| 0.97 \| 0.42, 2.10 \| 0.948 \| 1.1 \| 1.16 \| 0.53, 2.39 \| 0.700 \| 1.0 \|  \|  \|  \|  \| \| **Aβ1-40 (SD)** \| 0.86 \| 0.38, 1.80 \| 0.698 \| 7.2 \|  \|  \|  \|  \|  \|  \|  \|  \|  \|  \|  \|  \|  \|  \|  \|  \| 0.94 \| 0.44, 1.88 \| 0.868 \| 6.1 \| \| **Aβ1-42 (SD)** \| 1.02 \| 0.48, 2.18 \| 0.951 \| 7.0 \|  \|  \|  \|  \|  \|  \|  \|  \|  \|  \|  \|  \|  \|  \|  \|  \| 0.96 \| 0.47, 1.98 \| 0.908 \| 5.9 \| \| **p-tau181 (SD)** \| 1.27 \| 0.89, 3.78 \| 0.519 \| 2.1 \|  \|  \|  \|  \|  \|  \|  \|  \|  \|  \|  \|  \|  \|  \|  \|  \| 1.31 \| 0.82, 4.30 \| 0.626 \| 2.6 \| \| **p-tau217 (SD)** \| 1.85 \| 1.12, 2.88 \| **0.008** \| 2.2 \|  \|  \|  \|  \|  \|  \|  \|  \|  \|  \|  \|  \|  \|  \|  \|  \| 1.86 \| 1.14, 2.91 \| **0.012** \| 2.3 \| \| **Aβ1-42/1-40 (SD)** \|  \|  \|  \|  \| 0.91 \| 0.65, 1.29 \| 0.598 \| 1.1 \|  \|  \|  \|  \|  \|  \|  \|  \|  \|  \|  \|  \|  \|  \|  \|  \| \| **AT^181^term (SD)** \|  \|  \|  \|  \|  \|  \|  \|  \| 2.83 \| 1.43, 5.97 \| **0.004** \| 1.1 \|  \|  \|  \|  \|  \|  \|  \|  \|  \|  \|  \|  \| \| **AT^217^term (SD)** \|  \|  \|  \|  \|  \|  \|  \|  \|  \|  \|  \|  \| 2.05 \| 1.49, 2.88 \| **<0.001** \| 1.1 \|  \|  \|  \|  \|  \|  \|  \|  \| \| AIC \| 284 \|  \|  \|  \| 300 \|  \|  \|  \| 285 \|  \|  \|  \| 281 \|  \|  \|  \| 299 \|  \|  \|  \| 288 \|  \|  \|  \| \| BIC \| 327 \|  \|  \|  \| 331 \|  \|  \|  \| 316 \|  \|  \|  \| 313 \|  \|  \|  \| 327 \|  \|  \|  \| 307 \|  \|  \|  \| \| No. Obs. \| 382 \|  \|  \|  \| 382 \|  \|  \|  \| 382 \|  \|  \|  \| 382 \|  \|  \|  \| 388 \|  \|  \|  \| 382 \|  \|  \|  \| | | | | | | | | | | | | | | | | | | | | | | | | |

Multiple logistic regression for 12 months-POCD Stage 3

| **Models** | **1 - Clinical + BBM** | | | | **2 - Clinical + Aβ42/40** | | | | **3 – Clinical + AT^181^term** | | | | **4 – Clinical + AT^217^term** | | | | **5 – Clinical only** | | | | **6 – BBM only** | | | |
| --- | --- | --- | --- | --- | --- | --- | --- | --- | --- | --- | --- | --- | --- | --- | --- | --- | --- | --- | --- | --- | --- | --- | --- | --- |
| **Characteristic** | **OR** | **95% CI** | **p-value** | **VIF** | **OR** | **95% CI** | **p-value** | **VIF** | **OR** | **95% CI** | **p-value** | **VIF** | **OR** | **95% CI** | **p-value** | **VIF** | **OR** | **95% CI** | **p-value** | **VIF** | **OR** | **95% CI** | **p-value** | **VIF** |
| **(Intercept)** | 0.01 | 0.00, 0.80 | **0.044** |  | 0.01 | 0.00, 0.40 | **0.019** |  | 0.01 | 0.00, 0.36 | **0.016** |  | 0.02 | 0.00, 0.86 | **0.049** |  | 0.01 | 0.00, 0.24 | **0.009** |  | 0.07 | 0.05, 0.11 | **<0.001** |  |
| **Female sex** | 0.29 | 0.06, 0.95 | 0.068 | 1.1 | 0.31 | 0.07, 0.98 | 0.074 | 1.1 | 0.30 | 0.07, 0.93 | 0.064 | 1.1 | 0.30 | 0.06, 0.96 | 0.070 | 1.1 | 0.27 | 0.06, 0.87 | 0.051 | 1.1 |  |  |  |  |
| **Age** | 1.06 | 1.01, 1.12 | **0.034** | 1.2 | 1.07 | 1.02, 1.13 | **0.012** | 1.1 | 1.07 | 1.02, 1.13 | **0.008** | 1.1 | 1.06 | 1.01, 1.11 | **0.032** | 1.1 | 1.07 | 1.02, 1.13 | **0.007** | 1.1 |  |  |  |  |
| **CABG** | 0.09 | 0.01, 0.66 | **0.040** | 7.6 | 0.09 | 0.01, 0.59 | **0.031** | 7.9 | 0.08 | 0.01, 0.57 | **0.028** | 8.0 | 0.09 | 0.01, 0.57 | **0.026** | 7.2 | 0.10 | 0.01, 0.62 | **0.029** | 6.8 |  |  |  |  |
| **Valve surgery** | 0.14 | 0.01, 0.94 | 0.080 | 7.2 | 0.14 | 0.01, 0.93 | 0.079 | 7.4 | 0.13 | 0.01, 0.89 | 0.070 | 7.5 | 0.14 | 0.01, 0.86 | 0.064 | 6.7 | 0.17 | 0.02, 0.97 | 0.080 | 6.3 |  |  |  |  |
| **Other Surgery** | 0.98 | 0.32, 2.61 | 0.969 | 1.2 | 1.14 | 0.40, 2.90 | 0.796 | 1.1 | 1.12 | 0.40, 2.84 | 0.824 | 1.1 | 1.01 | 0.34, 2.63 | 0.990 | 1.1 | 1.10 | 0.39, 2.79 | 0.854 | 1.1 |  |  |  |  |
| **POD** | 1.98 | 0.78, 4.75 | 0.136 | 1.1 | 2.15 | 0.87, 5.03 | 0.085 | 1.1 | 2.26 | 0.93, 5.20 | 0.061 | 1.0 | 2.02 | 0.81, 4.77 | 0.115 | 1.0 | 2.31 | 0.96, 5.32 | 0.053 | 1.0 |  |  |  |  |
| **Aβ1-40 (SD)** | 1.17 | 0.47, 2.76 | 0.731 | 6.8 |  |  |  |  |  |  |  |  |  |  |  |  |  |  |  |  | 1.54 | 0.67, 3.34 | 0.289 | 6.4 |
| **Aβ1-42 (SD)** | 0.99 | 0.38, 2.59 | 0.982 | 7.0 |  |  |  |  |  |  |  |  |  |  |  |  |  |  |  |  | 0.75 | 0.32, 1.85 | 0.518 | 7.0 |
| **p-tau181 (SD)** | 0.40 | 0.06, 1.19 | 0.277 | 2.8 |  |  |  |  |  |  |  |  |  |  |  |  |  |  |  |  | 0.44 | 0.08, 1.09 | 0.275 | 3.7 |
| **p-tau217 (SD)** | 2.00 | 1.11, 3.84 | **0.027** | 2.4 |  |  |  |  |  |  |  |  |  |  |  |  |  |  |  |  | 2.09 | 1.22, 3.83 | **0.012** | 3.0 |
| **Aβ1-42/1-40 (SD)** |  |  |  |  | 0.86 | 0.56, 1.33 | 0.492 | 1.1 |  |  |  |  |  |  |  |  |  |  |  |  |  |  |  |  |
| **AT^181^term (SD)** |  |  |  |  |  |  |  |  | 1.12 | 0.47, 1.47 | 0.574 | 1.1 |  |  |  |  |  |  |  |  |  |  |  |  |
| **AT^217^term (SD)** |  |  |  |  |  |  |  |  |  |  |  |  | 1.60 | 1.04, 2.35 | **0.021** | 1.1 |  |  |  |  |  |  |  |  |
| AIC | 200 |  |  |  | 200 |  |  |  | 201 |  |  |  | 196 |  |  |  | 200 |  |  |  | 204 |  |  |  |
| BIC | 244 |  |  |  | 232 |  |  |  | 232 |  |  |  | 228 |  |  |  | 227 |  |  |  | 223 |  |  |  |
| No. Obs. | 382 |  |  |  | 382 |  |  |  | 382 |  |  |  | 382 |  |  |  | 388 |  |  |  | 382 |  |  |  |
| Abbreviations: Aβ40 = amyloid-beta 40, Aβ42 = amyloid-beta 42, AIC = Akaike information criterion, AT^181^term = amyloid- beta 40/ amyloid-beta42* p-tau181,  AT^217^term = amyloid- beta 40/ amyloid-beta42* p-tau181, BBM = blood biomarker, BIC = Bayesian information criterion, CABG = coronary bypass graft, CI = confidence interval,  Aβ1-42 = amyloid-β1-42, Aβ1-40 = amyloid-β40, AT^181^term = amyloid-β1-40/ 1-42*p-tau181, AT^217^term= amyloid-β1-40/ 1-42*p-tau217, POD = postoperative delirium, No. obs. = number of observations, p-tau181 = phosphorylated tau protein181, p-tau217 = phosphorylated tau protein 217,  VIF = variance inflation factor | | | | | | | | | | | | | | | | | | | | | | | | |
