# Supplementary material for: Early Alzheimer´s disease blood biomarkers are associated with a higher risk for postoperative long‐term cognitive decline: Insights from the FINDERI study
Source: Alzheimers Dement. 2026 Jul 14;22(7):e71631. doi: 10.1002/alz.71631 (PMC13368704; doi:10.1002/alz.71631)
Supplement: Supplementary file 6 — Supporting information [file ALZ-22-e71631-s003.docx]

Supplement Table 6: Multiple logistic regressions for POCD including ApoE4 geno- and proteotype as explaining variables

A. Multiple logistic regressions for POCD Stage 1

|  | Model 1 | | | | | Model 4 | | | | | Model 5 | | | | Model 6 | | | | |
| --- | --- | --- | --- | --- | --- | --- | --- | --- | --- | --- | --- | --- | --- | --- | --- | --- | --- | --- | --- |
| **Characteristic** | **OR** | **95% CI** | **p-value** | **GVIF** | **Adjusted GVIF**^1^ | **OR** | **95% CI** | **p-value** | **GVIF** | **Adjusted GVIF**^1^ | **OR** | **95% CI** | **p-value** | **VIF** | **OR** | **95% CI** | **p-value** | **GVIF** | **Adjusted GVIF**^1^ |
| **(Intercept)** | 0.58 | 0.06, 5.29 | 0.630 |  |  | 0.70 | 0.08, 6.26 | 0.746 |  |  | 0.36 | 0.05, 2.71 | 0.322 |  | 0.39 | 0.28, 0.53 | **<0.001** |  |  |
| **ApoE-e4 Genotype** |  |  |  | 2.6 | 1.3 |  |  |  | 2.5 | 1.3 |  |  |  |  |  |  |  | 2.7 | 1.3 |
| Non e4 | — | — |  |  |  | — | — |  |  |  |  |  |  |  | — | — |  |  |  |
| Heterozygous e4 | 0.90 | 0.45, 1.88 | 0.777 |  |  | 0.91 | 0.46, 1.85 | 0.779 |  |  |  |  |  |  | 0.89 | 0.45, 1.87 | 0.753 |  |  |
| Homozygous e4 | 0.18 | 0.00, 2.28 | 0.251 |  |  | 0.19 | 0.00, 2.32 | 0.262 |  |  |  |  |  |  | 0.16 | 0.00, 2.16 | 0.231 |  |  |
| AIC | 445 |  |  |  |  | 442 |  |  |  |  | 457 |  |  |  | 439 |  |  |  |  |
| BIC | 500 |  |  |  |  | 485 |  |  |  |  | 484 |  |  |  | 470 |  |  |  |  |
| No. Obs. | 378 |  |  |  |  | 378 |  |  |  |  | 388 |  |  |  | 378 |  |  |  |  |
| ^1^GVIF^[1/(2*df)] | | | | | | | | | | | | | | | | | | | |
| Abbreviations: CI = Confidence Interval, GVIF = Generalized Variance Inflation Factor, OR = Odds Ratio, VIF = Variance Inflation Factor | | | | | | | | | | | | | | | | | | | |

B. Multiple logistic regressions for POCD Stage 2

|  | Model 1 | | | | | Model 4 | | | | | Model 5 | | | | Model 6 | | | | |
| --- | --- | --- | --- | --- | --- | --- | --- | --- | --- | --- | --- | --- | --- | --- | --- | --- | --- | --- | --- |
| **Characteristic** | **OR** | **95% CI** | **p-value** | **GVIF** | **Adjusted GVIF**^1^ | **OR** | **95% CI** | **p-value** | **GVIF** | **Adjusted GVIF**^1^ | **OR** | **95% CI** | **p-value** | **VIF** | **OR** | **95% CI** | **p-value** | **GVIF** | **Adjusted GVIF**^1^ |
| **(Intercept)** | 0.14 | 0.01, 3.07 | 0.212 |  |  | 0.14 | 0.01, 2.94 | 0.208 |  |  | 0.03 | 0.00, 0.50 | **0.015** |  | 0.14 | 0.08, 0.22 | **<0.001** |  |  |
| **ApoE-e4 Genotype** |  |  |  | 3.0 | 1.3 |  |  |  | 2.9 | 1.3 |  |  |  |  |  |  |  | 3.0 | 1.3 |
| Non e4 | — | — |  |  |  | — | — |  |  |  |  |  |  |  | — | — |  |  |  |
| Heterozygous e4 | 1.29 | 0.38, 4.21 | 0.677 |  |  | 1.24 | 0.38, 3.99 | 0.717 |  |  |  |  |  |  | 1.42 | 0.44, 4.49 | 0.551 |  |  |
| Homozygous e4 | 0.00 | 0.00, 83,151 | 0.984 |  |  | 0.00 | 0.00, 3,263,570 | 0.984 |  |  |  |  |  |  | 0.00 | 0.00, 1,829,535 | 0.984 |  |  |
| **ApoE4, SD** | 0.90 | 0.28, 2.39 | 0.847 | 2.8 | 1.7 | 0.90 | 0.29, 2.35 | 0.840 | 2.9 | 1.7 |  |  |  |  | 0.80 | 0.26, 2.03 | 0.664 | 2.8 | 1.7 |
| AIC | 286 |  |  |  |  | 283 |  |  |  |  | 299 |  |  |  | 288 |  |  |  |  |
| BIC | 341 |  |  |  |  | 327 |  |  |  |  | 327 |  |  |  | 320 |  |  |  |  |
| No. Obs. | 378 |  |  |  |  | 378 |  |  |  |  | 388 |  |  |  | 378 |  |  |  |  |
| ^1^GVIF^[1/(2*df)] | | | | | | | | | | | | | | | | | | | |
| Abbreviations: CI = Confidence Interval, GVIF = Generalized Variance Inflation Factor, OR = Odds Ratio, VIF = Variance Inflation Factor | | | | | | | | | | | | | | | | | | | |

C. Multiple logistic regressions for POCD Stage 3

|  | Model 1 | | | | | Model 4 | | | | | Model 5 | | | | Model 6 | | | | |
| --- | --- | --- | --- | --- | --- | --- | --- | --- | --- | --- | --- | --- | --- | --- | --- | --- | --- | --- | --- |
| **Characteristic** | **OR** | **95% CI** | **p-value** | **GVIF** | **Adjusted GVIF**^1^ | **OR** | **95% CI** | **p-value** | **GVIF** | **Adjusted GVIF**^1^ | **OR** | **95% CI** | **p-value** | **VIF** | **OR** | **95% CI** | **p-value** | **GVIF** | **Adjusted GVIF**^1^ |
| **(Intercept)** | 0.01 | 0.00, 0.67 | **0.036** |  |  | 0.01 | 0.00, 0.68 | **0.038** |  |  | 0.01 | 0.00, 0.24 | **0.009** |  | 0.07 | 0.03, 0.12 | **<0.001** |  |  |
| **ApoE-e4 Genotype** |  |  |  | 2.9 | 1.3 |  |  |  | 2.6 | 1.3 |  |  |  |  |  |  |  | 2.7 | 1.3 |
| Non e4 | — | — |  |  |  | — | — |  |  |  |  |  |  |  | — | — |  |  |  |
| Heterozygous e4 | 1.23 | 0.29, 4.83 | 0.767 |  |  | 1.36 | 0.35, 5.03 | 0.647 |  |  |  |  |  |  | 1.64 | 0.42, 5.97 | 0.461 |  |  |
| Homozygous e4 | 0.00 | 0.00, 159,692,704,474 | 0.988 |  |  | 0.00 | 0.00, 2,582,495,445 | 0.988 |  |  |  |  |  |  | 0.00 | 0.00, 94,219,452,475,572,862,986 | 0.990 |  |  |
| **ApoE4, SD** | 1.37 | 0.41, 3.85 | 0.573 | 2.7 | 1.7 | 1.35 | 0.41, 3.61 | 0.579 | 2.6 | 1.6 |  |  |  |  | 1.08 | 0.35, 2.80 | 0.888 | 2.5 | 1.6 |
| AIC | 202 |  |  |  |  | 198 |  |  |  |  | 200 |  |  |  | 205 |  |  |  |  |
| BIC | 258 |  |  |  |  | 241 |  |  |  |  | 227 |  |  |  | 236 |  |  |  |  |
| No. Obs. | 378 |  |  |  |  | 378 |  |  |  |  | 388 |  |  |  | 378 |  |  |  |  |
| ^1^GVIF^[1/(2*df)] | | | | | | | | | | | | | | | | | | | |
| Abbreviations: Aβ1-40 = amyloid-beta 1-40, Aβ1-42 = amyloid-beta 1-42, AIC = Akaike information criterion, AT^181^term = amyloid- beta 1-40/ 1-42* p-tau181,  AT^217^term = amyloid- beta 1-40/ 1-42* p-tau181, BB = blood biomarker, BIC = Bayesian information criterion, CABG = coronary bypass graft, CI = confidence interval, GVIF = generalized variance inflation factor, OR = Odds Ratio, POD = postoperative delirium, No. obs. = number of observations, p-tau181 = phosphorylated tau protein181, p-tau217 = phosphorylated tau protein 217, SD = standard deviation, VIF = variance inflation factor | | | | | | | | | | | | | | | | | | | |

**Supplement Table 2A-C:**

Model 2 (Aβ1-42/1-40) was not included in multiple regressions with ApoE4 proteo-and genotype due to the high collinearity of the Aβ1-40 and Aβ1-42 values in multiple logistic regression models for multiple regression with ApoE4 genotype and proteotype to prevent multicollinearity (Supplement 1). The term AT^181^term was not included as a model (model 3) in multiple regressions with ApoE4 proteo-and genotype, as it does not prove to be a relevant predictor in multiple regression analysis without consideration of the ApoE geno- and proteotype.
