# Supplementary material for: Early Alzheimer´s disease blood biomarkers are associated with a higher risk for postoperative long‐term cognitive decline: Insights from the FINDERI study
Source: Alzheimers Dement. 2026 Jul 14;22(7):e71631. doi: 10.1002/alz.71631 (PMC13368704; doi:10.1002/alz.71631)
Supplement: Supplementary file 7 — Supporting information [file ALZ-22-e71631-s001.docx]

**Supplement Text: POCD classification strategy**

The definition of POCD stages relies on the MoCA scores at both baseline and the 12-month follow-up. A patient is classified as having POCD Stage 1, 2, or 3 based on the criteria below, where 'x' represents the specific threshold at each stage:

1. Their baseline MoCA score was ≥ x and their 1y-FU MoCA score is < x.
2. Their baseline MoCA score was already < x and their 1y-FU MoCA score dropped further (meaning: baseline score > 1y-FU score).

The specific values for 'x' defining each POCD stage are 26 for POCD Stage 1, 23 for Stage 2, and 21 for Stage 3.

As an example, a person with a baseline MoCA of 25 points and a 1y-FU MoCA of 23 points:

- would have POCD Stage 1 because their baseline score (25) was < 26 (Condition 2 is met, as 25 > 23).
- would not have POCD Stage 2 because their 12-month follow-up score (23) is not < 23, and their baseline score (25) is not < 23.
- would not have POCD Stage 3 because their 12-month follow-up score (23) is not < 21, and their baseline score (25) is not < 21.

**Abbreviations:** 1y-FU = 1 year follow-up, MoCA = Montreal Cognitive Assessment, POCD = postoperative cognitive dysfunction
